# Why diabetes matters in dementia studies: Failure to account for diabetes status masks regional mitochondrial DNA copy number changes in human hippocampus, amygdala, and cerebellum in Alzheimer's disease

**DOI:** 10.1002/alz.71870

**Published:** 2026-10-20

**Authors:** Aakruti Kaikini, Anqi Shi, Paul Francis, Russell Swerdlow, Claire Troakes, Jaimee Kennedy, Alan Hodgkinson, Afshan N. Malik

**Affiliations:** ^1^ King's College London, Diabetes and Obesity, School of Cardiovascular Medicine and Metabolic Sciences Faculty of Life Sciences and Medicine London England; ^2^ Dept of Clinical and Biomedical Sciences University of Exeter University of Exeter Medical School London England; ^3^ University of Kansas Alzheimer's Disease Research Center University of Kansas School of Medicine Fairway Kansas USA; ^4^ Neurodegenerative Diseases Brain Bank, Department of Clinical and Basic Neuroscience, Institute of Psychiatry, Psychology &Neuroscience King's College London London England; ^5^ Department of Medical and Molecular Genetics, School of Basic and Medical Biosciences King's College London London England

**Keywords:** Alzheimer's disease, biobank, co‐morbidity, diabetes, mitochondrial DNA, post‐mortem brain

## Abstract

**INTRODUCTION:**

Diabetes is a major risk factor for Alzheimer's disease (AD), and both diseases involve mitochondrial dysfunction. Changes in mitochondrial DNA (mtDNA) can influence energy metabolism, cellular signaling, and epigenetic programming, thereby affecting cellular health. We hypothesized that AD is associated with reduced mtDNA copy number (mtDNA‐CN) in vulnerable brain regions, and that diabetes modifies these changes.

**METHODS:**

MtDNA‐CN was quantified by absolute quantitative polymerase chain reaction (qPCR) in post‐mortem hippocampus, amygdala, and cerebellum from cognitive controls (CCs), AD, and diabetes groups (CC+D, AD+D; *N* = 66–77).

**RESULTS:**

MtDNA‐CN differed by region, being highest in amygdala, intermediate in hippocampus, and lowest in cerebellum (503, 421, and 160 copies/cell; *p* < 0.0001). Pooled analyses showed modestly lower mtDNA‐CN in AD. However, inclusion of diabetes appeared to mask differences. Donors with diabetes (CC+D and AD+D combined) had higher mtDNA‐CN than non‐diabetic (CC and AD combined) in hippocampus (+37.5%; *p* = 0.0012), amygdala (+30.6%; *p* = 0.0057), and cerebellum (+48.5%; *p* = 0.0002). This diabetes‐associated increase was also present within AD, where AD+D had higher mtDNA‐CN than AD in all regions. Removal of diabetic donors showed that AD was associated with a 19%–30% lower mtDNA‐CN, reaching significance in the amygdala and cerebellum after correction for multiple testing.

**DISCUSSION:**

We show that AD is associated with multiregional mtDNA‐CN loss most clearly in donors without diabetes, whereas diabetes is associated with higher mtDNA‐CN and can mask AD‐related mitochondrial changes. The functional significance of higher mtDNA‐CN in the diabetic brain remains unclear, but evidence that diabetes acts as an effect modifier has important implications for dementia studies.

## Highlights

- Diabetes was consistently associated with higher mitochondrial DNA copy number (mtDNA‐CN) in post‐mortem human hippocampus, amygdala, and cerebellum, independent of cognitive status.
- Pooled analyses showed modestly lower mtDNA‐CN in AD which were not significant after correcting for multiple testing.
- Analysis of non‐diabetic cases only showed that AD was associated with a 19%–30% lower mtDNA‐CN, reaching significance in the amygdala and cerebellum after correction for multiple testing.
- We show that diabetes acts as an effect modifier of mitochondrial readouts in AD brain, suggesting that diabetes may alter mitochondrial biology in the human brain through mechanisms distinct from those underlying non‐diabetic AD.

## 1 BACKGROUND

Alzheimer's disease (AD), the leading cause of dementia, affects >57 million people worldwide, is predicted to rise to >150 million by 2050.^1^ AD is increasingly recognized as a heterogeneous disorder, with multiple contributing mechanisms and more than 14 known modifiable risk factors.^2^, ^3^ Diabetes mellitus, a metabolic disorder characterized by chronic hyperglycemia, affects >550 million individuals worldwide, increases the risk of developing AD by 40%–60%.^4^, ^5^

Diabetes and AD share several pathological features including insulin resistance, mitochondrial dysfunction, chronic inflammation, and oxidative stress, suggesting that diabetes may accelerate neurodegenerative changes.^6^, ^7^ In addition to shared mechanisms, structural and molecular differences have been reported between diabetic and non‐diabetic AD.^8^, ^9^, ^10^ Positron emission tomography (PET) studies show a distinct clinical profile in dementia patients with diabetes, characterized by reduced amyloid burden and prominent tau pathology.^11^ This suggests that clinically defined AD cohorts may comprise metabolically distinct subgroups with differing vascular, inflammatory, and bioenergetic contributions, potentially requiring tailored therapeutic strategies.

The human brain has high energy needs, largely met through oxidative phosphorylation via the electron transport chain, whose critical subunits are encoded by mitochondrial DNA (mtDNA). mtDNA is used widely as a biomarker of mitochondrial content; we have previously shown that some human brain regions can have >5000 mtDNA copies per cell (mtDNA‐CN), making it a highly abundant molecule.^12^ Changes in mtDNA‐CN can influence energy metabolism, cellular signaling, and epigenetic programming, thereby affecting cellular health.^13^ Reduced mtDNA‐CN has been reported in several metabolic diseases, largely in the periphery, including diabetes, AD, and cardiovascular disease.^14^, ^15^, ^16^, ^17^ Lower mtDNA‐CN is generally associated with higher disease risk, supporting the idea that mtDNA‐CN depletion may result in compromised mitochondrial function, loss of bioenergetic flexibility, and cellular damage.^18^, ^19^

**RESEARCH IN CONTEXT**

1. **Systematic review**: Studies assessing mitochondrial DNA copy number (mtDNA‐CN) in human post‐mortem Alzheimer's disease (AD) brain report inconsistent findings, with reductions, no change or region‐specific effects described across cohorts. A key limitation is that these studies did not account for or analyze diabetes status, a major metabolic risk factor for dementia known to influence mitochondrial function. To date, only our group's prior work has explicitly stratified mtDNA‐CN analysis by diabetes status in post‐mortem parietal cortex, showing that diabetes is associated with higher mtDNA‐CN. The relevant studies are appropriately cited. Overall, the literature lacks systematic evaluation of how metabolic comorbidities may modify mitochondrial readouts in AD brain.
2. **Interpretation**: Our findings demonstrate that diabetes and AD exert opposing effects on mtDNA‐CN in three regions of human brain. When diabetes status is not considered, mtDNA‐CN shows modest reduction in AD, indicating that the opposing effects obscure AD‐related reductions in mtDNA‐CN, providing a compelling explanation for the inconsistent findings reported in the literature. When AD cases are stratified by diabetes status, the reduction in mtDNA‐CN becomes significant in non‐diabetic AD group. Diabetes‐stratified analysis revealed stronger and statistically significant reductions in mtDNA‐CN in non‐diabetic AD, supporting the view that diabetes may be an effect modifier rather than a background covariate in AD studies. These findings highlight that metabolic heterogeneity within AD cohorts can substantially distort molecular readouts.
3. **Future directions**: Large biobanks should prioritize systematic documentation of metabolic parameters, particularly diabetes status, to minimize confounding in molecular and neuropathological analysis. Future studies investigating mitochondrial function and therapeutic and biomarker development should also incorporate diabetes status as a core analytical variable. Accounting for metabolic stratification may reveal subgroup‐specific disease mechanisms and advance precision‐medicine approaches. Finally, mechanistic studies are needed to determine whether AD in individuals with diabetes reflects distinct underlying pathophysiology compared to non‐diabetic AD, and to understand the functional impact of the increased mtDNA‐CN in the diabetic brain.

Studies examining mtDNA‐CN in the post‐mortem human AD brain have produced inconsistent results. Some report reduced mtDNA‐CN in cortical and hippocampal regions, with cerebellum typically unchanged. Whereas, large scale studies found no changes, leaving unresolved whether AD brain shows regional or general loss of mitochondrial content.^20^, ^21^, ^22^, ^23^, ^24^, ^25^ Of note, most studies did not report the diabetic status of the donors, often due to incomplete clinical records. A previous study from our group is the only published study examining diabetic and non‐diabetic AD.^12^ We observed reduced mtDNA‐CN in the parietal cortex of non‐diabetic AD, with no change in frontal cortex or cerebellum. Unexpectedly, diabetic donors exhibited higher mtDNA‐CN regardless of cognitive status in all three regions.^12^.This contrasted with our original expectation that diabetes would exacerbate AD‐related mitochondrial changes and instead suggested that diabetes and AD may exert opposing effects on brain mtDNA‐CN.

Given the high prevalence of diabetes, affecting 28.8% of adults ≥65 years of age and reported in 6.0%–34.6% among individuals with AD, diabetes is likely a common comorbidity in AD.^26^, ^27^, ^28^ Therefore, confirming whether regional or global loss of brain mitochondrial content is implicated in AD, and whether diabetic AD shows the same or different trends is important, as any differences may have implications for treatment strategies.

AD affects different brain regions, and vulnerability and clinical manifestations vary by region. For this study, we selected three brain regions with differential relevance to AD and diabetes: the hippocampus,^29^ one of the earliest regions impacted and essential for spatial memory and learning; the amygdala,^30^ which undergoes early atrophy linked to emotional and neuropsychiatric symptoms; and the cerebellum,^31^, ^32^ traditionally considered spared in AD but affected by diabetes and increasingly recognized for roles in cognition and insulin signaling. Although prior studies report mtDNA‐CN reductions in human hippocampus and no changes in cerebellum, mtDNA‐CN has never been measured in human amygdala. Furthermore, aside from diabetic cerebellar mtDNA‐CN reported in our earlier study,^12^ mtDNA‐CN in hippocampus and amygdala of diabetic individuals has never been reported.

We therefore hypothesized that AD is associated with region‐specific loss of mitochondrial content, measured as mtDNA‐CN, primarily affecting the early‐vulnerable hippocampus and amygdala. By stratifying donors according to AD and diabetes status, we aimed to determine whether diabetes modifies mtDNA‐CN in a way that may help explain heterogeneity within AD.

## 2 METHODS

### 2.1 The aims, design, and setting of the study

This cross‐sectional lab‐based study aimed to quantify mtDNA‐CN in three post‐mortem human brain regions: hippocampus, amygdala, and cerebellum, and determine if diabetes and AD, separately or in combination, is associated with altered mtDNA levels.

### 2.2 Ethical approval and informed consent

Project and ethical approval were obtained from the London Neurodegenerative Diseases Brain Bank (REC Reference 23/WA/0124). Post‐mortem tissue was handled, stored, used, and disposed as per the Human Tissue Act 2004. Written informed consent from the donors and/or their relatives as approporiate was obtained by the London Neurodegenerative Disease Brain Bank.

### 2.3 Human post‐mortem brain specimens

Samples for this study were selected based on the following predefined inclusion and exclusion criteria.

#### 2.3.1 Inclusion criteria

- Confirmed diagnosis or absence of AD based on combined clinical and neuropathological assessment.
- Confirmed diagnosis or absence of diabetes mellitus based on documented clinical assessment.
- Availability of frozen tissue suitable for molecular analysis from the same donor.

#### 2.3.2 Exclusion criteria

Samples were excluded if they showed significant history of other types of dementia including Parkinson's disease, frontal temporal dementia, or vascular dementia. All final group allocations and inclusion/exclusion decisions were made prior to laboratory analysis, and mtDNA‐CN was determined blinded to prevent bias.

### 2.4 Brain region sampling and processing

Samples had been processed according to the London Neurodegenerative Diseases Brain Bank brain bank's standard anatomic dissection procedures, applied uniformly across donors. Hippocampal tissue was specifically from the CA1 subregion, whereas subregion information for amygdala and cerebellum was not recorded. Hippocampal and amygdala tissue was obtained from 77 donors, whereas cerebellar tissue was available for 66 donors. All tissues were fresh‐frozen shortly after dissection and stored at −80°C until DNA extraction, with freeze–thaw cycles minimized to preserve integrity. Table 1 summarizes demographic information of cases used in the study.

**TABLE 1:** Demographic and clinical characteristics of cases included in the study.

Characteristic	CC	CC+D	AD	AD+D	Total cohort
Subjects, *n*	20	18	20	19	77
Age, years^a^	76.5 ± 7.4	78.9 ± 14.2	79.6 ± 8.4	81.5 ± 7.3	79.1 ± 9.6
PM delay, hours^a^	42.7 ± 18.2	42.4 ± 21.5	50.5 ± 22.2	46.7 ± 23.0	45.7 ± 21.1
Sex (female: male)	10:10	10:8	12:8	9:10	41:36
Diabetes status	No	Yes	No	Yes	NA
Braak stage	0–2	0–2	5–6	5–6	NA

^a^ Values shown are mean  ±  standard deviation.

Abbreviations: AD, non‐diabetic Alzheimer's disease; AD+D, diabetic Alzheimer's disease; CC, non‐diabetic non‐cognitively impaired; CC+D, diabetic non‐cognitively impaired; NA, not applicable; n, number of cases; PM, post‐mortem.

### 2.5 Quantification of mitochondrial DNA copy number

Total DNA was isolated using the QIAamp Fast DNA Tissue Kit, (Qiagen, UK), using the manufacturer's protocol optimized for brain tissue as follows. Tissue (≈20 mg) was homogenized in the buffer using a Tissue Lyser II (Qiagen, UK) for 30 s at 24 MHz. Homogenates were incubated in a thermomixer (Eppendorf, UK) at 1000 rpm for 10 min at 56°C with proteinase K. The resulting lysate was applied to the column and subjected to a series of washes with the provided wash buffers. DNA was eluted (50 µL elution buffer), concentration was determined by NanoDrop (Labtech International, UK), adjusted to a concentration of 10 ng/µL, and used in triplicate as a template with primer sequences targeting a unique region of human mtDNA (hMito) and the human nuclear gene beta‐2‐microglobulin (hB2M) (see Table S1 for primer sequences). Each 10 µL reaction consisted of 8 µL Master Mix (5 µL 2x Quantifast SYBR Green Master Mix, Qiagen; 500 nM forward and reverse primer; made up to volume with RNAase‐free water) and 2 µL DNA template. Samples were loaded onto a 96‐well plate in triplicate alongside a standard curve consisting of a serial dilution of 10^7^ to 10^3^ copies/2 µL of primer‐specific polymerase chain reaction (PCR) amplicons. All reactions were performed using the LightCycler 96 Real‐Time PCR System (Roche Diagnostics, Switzerland). Absolute mtDNA‐CN was calculated using the standard curve as a ratio of mitochondrial (hMito) to nuclear (hB2M) targets, representing cellular mtDNA content as described previously (MtDNA‐CN).^12^, ^33^

### 2.6 Statistical analysis

Statistical analyses were performed using GraphPad Prism software (Version 10.3.0) and R (Version 4.4.3).

For each brain region, outliers were defined using the interquartile range (IQR) method. The first quartile (Q1) represents the 25th percentile of the data, and the third quartile (Q3) represents the 75th percentile. The IQR is calculated as Q3–Q1. Values greater than Q3 + 3 × IQR or less than Q1–3 × IQR were classified as extreme outliers.

Group comparisons were performed using one‐tailed Student's *t*‐test when equal variances were confirmed by *F*‐test or Welch's *t*‐tests when variances were unequal. Significance thresholds used after correcting for multiple testing were: *p < 0.0167, ** *p* < 0.0033, ***p < 0.00033. Sex differences in mtDNA‐CN were assessed using Mann–Whitney *U* test, and Pearson correlation analyses were used to evaluate associations of mtDNA‐CN with age and post‐mortem interval (PMI).

Although data were initially available for 66 cases across all three brain regions, two cases were excluded following predefined outlier assessment, resulting in 64 cases in the final analytical dataset (shown below in results). We also performed inter‐regional analysis in the 64 donors with complete dataset available for hippocampus, amygdala, and cerebellum. Relationships between pairs of brain regions were examined using Spearman correlation analysis. Partial Spearman correlations were also performed to determine whether these pairwise relationships remained after controlling for the third brain region. To characterize multiregional patterns of mtDNA‐CN, hierarchical clustering was applied to *z*‐scored mtDNA‐CN values using Ward's minimum variance method. The resulting data‐driven clusters were designated as low, moderate, or very high, and samples were arranged according to cluster membership for visualization. Differences in cluster distribution across the four disease groups were assessed using a chi‐square test of independence. Principal component analysis (PCA) was performed on *z*‐score standardized mtDNA‐CN values to identify the main patterns of variation across the three regions. Intraclass correlation coefficient (ICC) analysis was used to assess the consistency of mtDNA‐CN across the three regions within the same individual. Kendall's coefficient of concordance (W) was used to determine whether the relative order of the three brain regions was similar across individuals. These analyses were first carried out in the whole dataset and then repeated after stratification into the four disease groups.

## 3 RESULTS

The study utilized post‐mortem brain samples from three regions of the human brain, namely hippocampus, amygdala, and cerebellum. Hippocampus and amygdala were available for all 77 cases, whereas cerebellum was available for 66 cases. Subjects were categorized as having no cognitive impairment (*n*  = 38) or AD (*n*  =  39), based on in‐life diagnosis and/or Braak neuropathology.^34^ Using their cognitive and diabetes status, the subjects were further categorized into four groups: No diabetes, cognitive controls with a Braak score of 0–2 (CC, *n* = 20); Diabetes and cognitive controls with a Braak score of 0–2 (CC+D, *n* = 18); AD and no diabetes, with a Braak score of 5–6 (AD, *n* = 20); Diabetes and AD with a Braak score of 5–6 (AD+D, *n* = 19) (Table 1). Table S2 lists the demographics, clinical characteristics, and mtDNA‐CN of the individual donors for the three brain regions.

Table S3 (A–C) show the raw technical replicate measurements for mitochondrial (hMito) and nuclear (hB2M) copy numbers for each sample from the hippocampus, amygdala, and cerebellum, respectively. For both targets, three technical replicates were generated (Mito 1–3 and B2M 1–3), with the percentage variation across replicates calculated. From these values, average copy numbers for hMito and hB2M were derived, and used to calculate the average MtDNA‐CN.

As determined by the IQR method described, two mtDNA‐CN values for cerebellum (452.0 and 704.6) exceeded the defined outlier thresholds (Q1–3 × IQR or Q3 + 3 × IQR) and were therefore excluded from subsequent analyses resulting in *n* = 64 for cerebellum.

### 3.1 Regional variation in mtDNA‐CN in the three brain regions

We observed regional differences in mtDNA‐CN across the three brain regions examined (Figure 1A). The amygdala contained the highest mtDNA‐CN, ranging from ≈200 to 1100 copies per cell (mean 503 copies/cell), followed by the hippocampus, which ranged from about 63 to 1110 copies per cell (mean 421 copies/cell). The cerebellum had the lowest mtDNA‐CN, ranging from ≈38 to 376 copies per cell (mean 160 copies/cell). Statistical analysis confirmed that MtDNA‐CN in cerebellum was significantly lower (*p* < 0.0001) than both amygdala and hippocampus mtDNA‐CN levels, whereas no significant difference was observed between the hippocampus and amygdala. In the whole dataset (*n* = 64), the dominant regional hierarchy was amygdala > hippocampus > cerebellum, observed in 40 of 64 individuals (62.5%), followed by hippocampus > amygdala > cerebellum in 21 of 64 individuals (32.8%) (Figure 1B). Spearman correlation analysis of the whole dataset showed a moderate positive correlation between hippocampus and amygdala (*ρ* = 0.48, *p* = 0.0001) and a weaker but significant positive correlation between hippocampus and cerebellum (*ρ* = 0.35, *p* = 0.005). In contrast, the correlation between amygdala and cerebellum was weak and did not reach significance (*ρ* = 0.24, *p* = 0.053) (Figure 1C). To further examine whether these relationships were direct or indirect, partial correlation analysis was performed (Figure 1C). The hippocampus–amygdala correlation remained strong after controlling for cerebellum (partial *ρ* = 0.44, *p* = 0.0003), and the hippocampus–cerebellum correlation also remained significant after controlling for amygdala (partial *ρ* = 0.28, *p* = 0.029). However, the amygdala–cerebellum correlation was lost after controlling for hippocampus (partial *ρ* = 0.09, *p* = 0.48). These findings suggest that hippocampus has independent relationships with both amygdala and cerebellum, whereas the apparent amygdala–cerebellum association is largely explained by their shared relationship with hippocampus.

**FIGURE 1:**
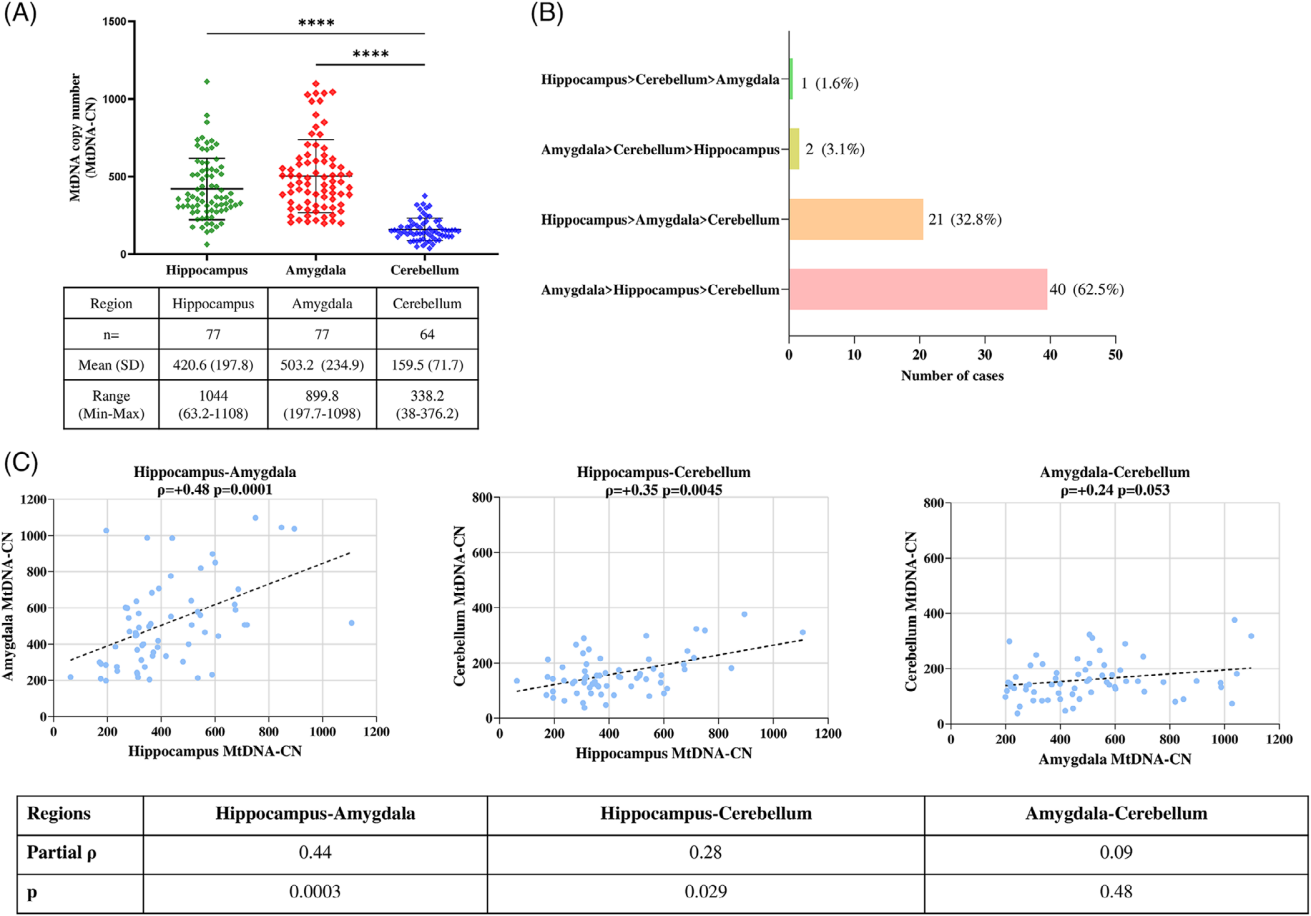
Regional differences and correlation between mitochondrial DNA copy number (mtDNA‐CN) in three regions of human post‐mortem brain. (A) mtDNA‐CN was measured in hippocampus (*n* = 77), amygdala (*n* = 77), and cerebellum (*n* = 64) of the human post‐mortem brain using absolute quantification by real‐time quantitative polymerase chain reaction (qPCR), with values expressed as ratio of mitochondrial to nuclear genome. Each datapoint represents mtDNA‐CN for an individual case, with data shown as mean ± standard deviation (SD). Group sizes (*n*) and descriptive data (mean, SD, range, minimum, and maximum) shown in the table below the graph. Statistical analysis was performed using the Kruskal–Wallis test followed by Dunn's multiple comparisons test; ****p < 0.0001. (B) Patterns of regional mtDNA‐CN levels in hippocampus, amygdala, and cerebellum within the same individual. The panel displays the frequency of the four possible ranking orders, indicating which region has higher or lower mtDNA‐CN relative to the others. (C) Correlation of mtDNA‐CN between brain region pairs. Scatterplots show pairwise comparisons of mtDNA‐CN between hippocampus–amygdala, hippocampus–cerebellum, and amygdala–cerebellum. Spearman's correlation coefficient (*ρ*) and corresponding *p*‐value are shown on each graph. A linear regression line is included for visualization. Partial correlation analysis, controlling for mtDNA‐CN in the third region, was also performed for each region pair, with partial ρ and *p*‐values shown in the table below. Correlations involving hippocampus remained significant after adjustment, whereas the amygdala–cerebellum association was not significant.

### 3.2 mtDNA‐CN shows no correlation with age, post‐mortem interval (PMI), and sex

MtDNA‐CN was compared between males and females in each brain region using Mann–Whitney *U* test. No significant sex differences were observed in any of the regions examined (Figure 2A). In subgroup analyses by diagnosis and diabetes status, hippocampus MtDNA‐CN showed sex‐associated difference within the CC group (*p* = 0.031), but this did not survive the regional multiple‐comparison threshold (*p* < 0.0167). No other sex‐associated differences were observed across the remaining group‐by‐region comparisons (Table S4).

**FIGURE 2:**
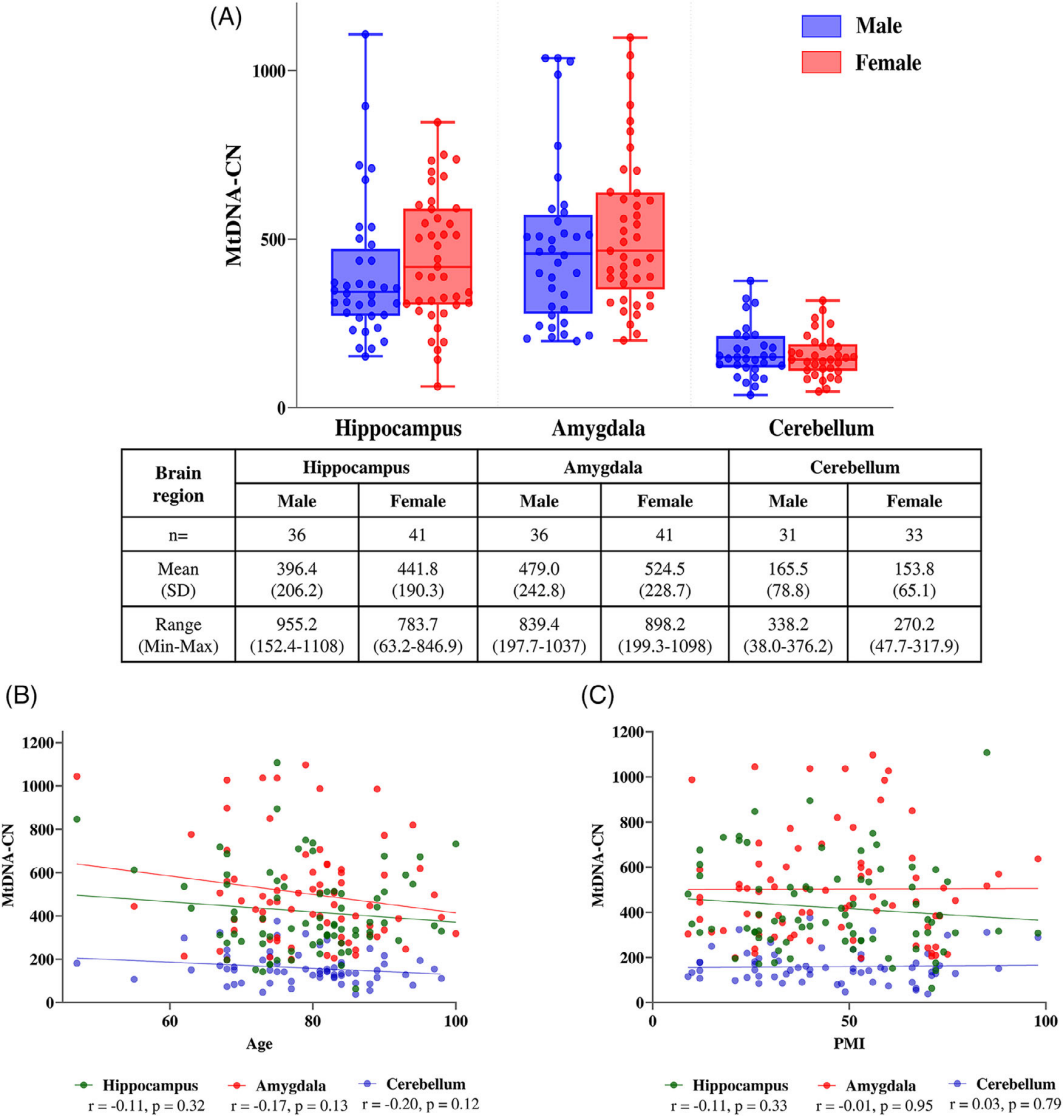
Sex, age, and post‐mortem interval do not influence mitochondrial DNA (mtDNA) copy number (mtDNA‐CN) in human brain. mtDNA‐CN in hippocampus (*n* = 77), amygdala (*n* = 77), and cerebellum (*n* = 64) was examined in relation to (A) biological sex, (B) age, and (C) post‐mortem interval in each of the three brain regions. (A) mtDNA‐CN across the three brain regions is stratified by sex (blue, male; red, female). Each dot represents mtDNA‐CN for an individual sample; boxplots display the median and interquartile range. No significant differences were observed between males and females in any brain region (Mann–Whitney *U* test). (B–C) Associations between mtDNA‐CN and either age (B) or post‐mortem interval (PMI) (C) across all samples are shown. Each dot represents mtDNA‐CN for an individual sample, with colors indicating brain region as shown. Pearson correlation coefficients (*r*) and corresponding *p*‐values are shown on each panel, and a simple linear regression line is included for visualization No significant associations were observed between mtDNA‐CN and either age or PMI in any brain region (*p* > 0.05).

Pearson correlation analyses were then conducted to examine the relationship of mtDNA‐CN with age and PMI in the amygdala, hippocampus, and cerebellum. No significant correlations were detected between mtDNA‐CN and either age (Figure 2B) or PMI (figure 2C) in any brain region. Together, these findings indicate that sex, age, and PMI did not influence mtDNA‐CN levels in this study.

### 3.3 mtDNA‐CN is reduced in AD and increased in diabetes

In all three brain regions, comparison of all AD subjects (AD and AD+D) versus all cognitive controls (CC and CC+D) showed modest reductions of 16%–19% of mtDNA‐CN (Figure 3A, Table 2). Because the same AD versus control comparison was performed separately in the three brain regions, three related statistical tests were conducted. To account for multiple testing and reduce the likelihood of false positive findings, a Bonferroni correction was applied whereby the conventional significance threshold (*p* < 0.05) was divided by the number of tests performed (0.05/3), resulting in a corrected significance threshold of *p* < 0.0167. Results with *p* < 0.05 but above this corrected threshold were therefore interpreted as nominally significant only. Nominal significance (*p* < 0.05) was observed in the hippocampus and amygdala (Figure 3A, Table 2); however, these did not survive after correcting for the number of brain regions tested (*p* > 0.0167 in all regions). The mean AD to CC ratio across all three regions was ≈0.83, indicating a similar magnitude of reduction in each region, thereby suggesting a biologically consistent effect.

**FIGURE 3:**
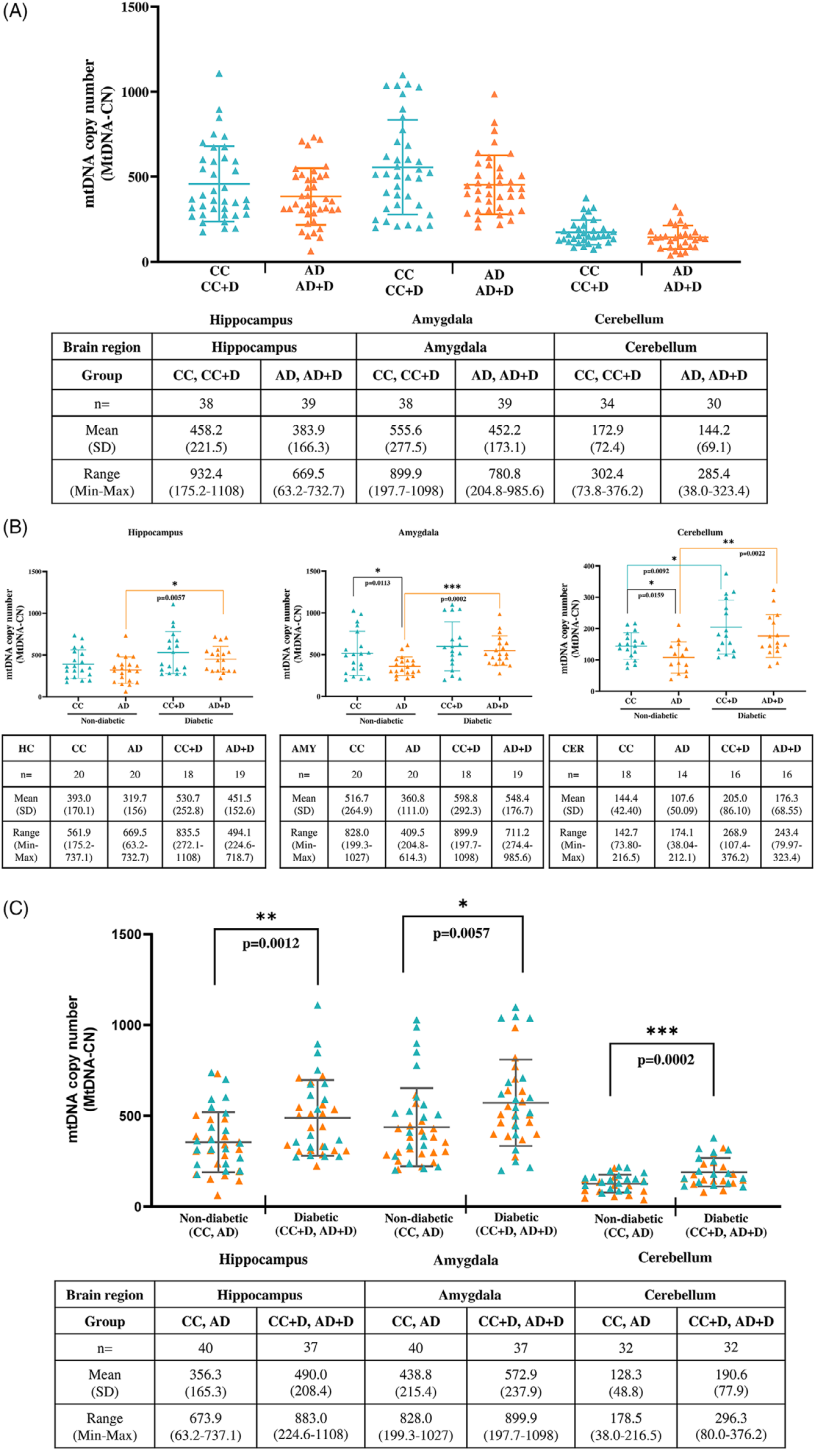
Mitochondrial DNA copy number (mtDNA‐CN) in three regions of human brain changes in Alzheimer's disease (AD) and diabetes (D). mtDNA‐CN in (A) all AD cases (AD and AD+D) versus all cognitive controls (CC, CC+D); (B) the four groups (CC, AD, CC+D, and AD+D); (C) non‐diabetic cases (CC and AD) versus diabetic cases (CC+D and AD+D). CC and CC+D shown as green triangles, and AD and AD+D shown as orange triangles. Each point represents the mtDNA‐CN of an individual sample. Horizontal bars indicate group means; error bars indicate standard deviation (SD). Group sizes (*n*) and descriptive data (mean, SD, range, minimum and maximum) are shown as tables below each figure panel. The *p*‐values were determined using student *t*‐test when variances were equal or Welch's *t*‐test when variances were unequal. To control for multiple testing across three brain regions, the nominal *p*‐value threshold (< 0.05) was divided by 3. Significance thresholds after correcting for multiple testing: *p < 0.0167; ** *p* < 0.0033, ***p < 0.00033. Only corrected‐significant comparisons are annotated on the graphs. HC, hippocampus; AMY, amygdala; CER, cerebellum; CC, non‐diabetic cognitive controls; CC+D, diabetic cognitive controls; AD, non‐diabetic Alzheimer's disease; AD+D, diabetic Alzheimer's disease.

**TABLE 2:** Summary of *p*‐values for each comparison for the three brain regions investigated.

Comparison	*p*‐values		
	Hippocampus	Amygdala	Cerebellum
CC, CC+D versus AD, AD+D (Figure 3A)	0.0498	0.0275^#^	0.0555
CC versus AD (Figure 3B)	0.0819	0.0113^#^	0.0159
CC+D versus AD +D (Figure 3B)	0.1307^#^	0.2666^#^	0.1526
CC versus CC+D	0.0272	0.1849	0.0092^#^
AD versus AD+D	0.0057	0.0002	0.0022
Non‐diabetic (CC+AD) versus diabetic (CC+D, AD+D) (Figure 3C)	0.0012	0.0057	0.0002^#^

*Note*: Group comparisons were carried out using either Student's *t*‐test (when variances were equal) or Welch's *t*‐test (when variances were unequal, values marked with #).

However, when restricting analysis to non‐diabetic subjects, stronger reductions of between 19% and 30% were observed in AD cases compared to CC cases (Figure 3B, Table 2) with mean ratios of ≈0.81 in hippocampus, 0.70 in amygdala, and 0.74 in cerebellum. Two brain regions, amygdala and cerebellum, reached statistical significance, even after correction for multiple testing despite smaller sample sizes (*p* < 0.0167, Table 2). By contrast, in subjects with diabetes, AD+D cases showed only small reductions of 8%–15% compared to CC+D (in all regions, *p* > 0.05, Figure 3B, Table 2). To further assess the effect of diabetes status within diagnostic groups, we compared CC with CC+D and AD with AD+D within each brain region. Within cognitive controls, diabetes was associated with higher mtDNA‐CN (Figure 3B, Table 2), with levels 42% higher in the cerebellum, reaching corrected significance (*p* = 0.0092) and showing a nominal increase of 35% in hippocampus (*p* = 0.0272). In contrast, the 16% increase observed in amygdala did not reach statistical significance. Within AD cases, diabetes was associated with significantly higher mtDNA‐CN across all three regions (Figure 3B, Table 2), with levels 41% higher in hippocampus (*p* = 0.0057), 52% higher in amygdala (*p* = 0.0002), and 64% higher in the cerebellum (*p* = 0.0022). These findings support diabetes as a strong modifier of mtDNA‐CN, particularly in AD.

Finally, and across the entire cohort, diabetes itself was associated with the opposing direction of change, showing highly significant increases in mtDNA‐CN of 30%–49% across the three regions, which remained highly significant after correction for multiple testing (*p* < 0.0167 in all regions, Figure 3C, Table 2). In individuals with diabetes, mean mtDNA‐CN was 37.5% higher in hippocampus, 30.6% higher in amygdala, and 48.5% higher in cerebellum compared to the non‐diabetic cases, corresponding to mean ratios of ≈1.38 in hippocampus (*p* = 0.0012), 1.31 in amygdala (*p* = 0.0057), and 1.49 in cerebellum (*p* = 0.0002), respectively. Notably, AD alone was linked to lower mtDNA‐CN (Figure 3B), whereas diabetes was linked to higher mtDNA‐CN (Figure 3C), showing opposing effects that effectively cancelled each other out when diabetes status was not considered (Figure 3A).

### 3.4 mtDNA‐CN levels in different brain regions of the same individual

Because we had used three brain regions from the same individuals (*n* = 64, Figure 4A), we examined the similarities in mtDNA‐CN levels across the three regions to determine any group patterns using hierarchical cluster analysis using Ward's method on *z*‐scored mtDNA‐CN. This resulted in identification of three distinct clusters: low, moderate, and very high (Figure 4B). Cluster membership was found to be significantly associated with disease group (*χ* ^2^ = 24.2, *df* = 6, *p* = 0.0005). The lower cluster (*n* = 23) was characterized by uniformly below average mtDNA‐CN across all three regions (mean z‐scores: hippocampus = −0.67, amygdala = −0.76, cerebellum = −0.69) and was enriched for AD (11/23, 48%) and CC (7/23, 30%), with 4 AD+D (17%) and only 1 CC+D (4%). The moderate cluster (*n* = 36) was characterized by near‐average mtDNA‐CN across all regions (mean *z*‐scores: hippocampus = +0.16, amygdala = +0.39, cerebellum = +0.12) and was the largest cluster showing a representation from all four disease groups (CC = 11, CC+D = 11, AD = 3, AD+D = 11). The very high cluster (*n* = 5) was characterized by markedly elevated mtDNA‐CN, particularly in hippocampus and cerebellum (mean z‐scores: Hippocampus = +1.95, amygdala = +0.70, cerebellum = +2.33). This cluster was dominated by CC+D (4/5, 80%), with 1AD+D and no non‐diabetic donors.

**FIGURE 4:**
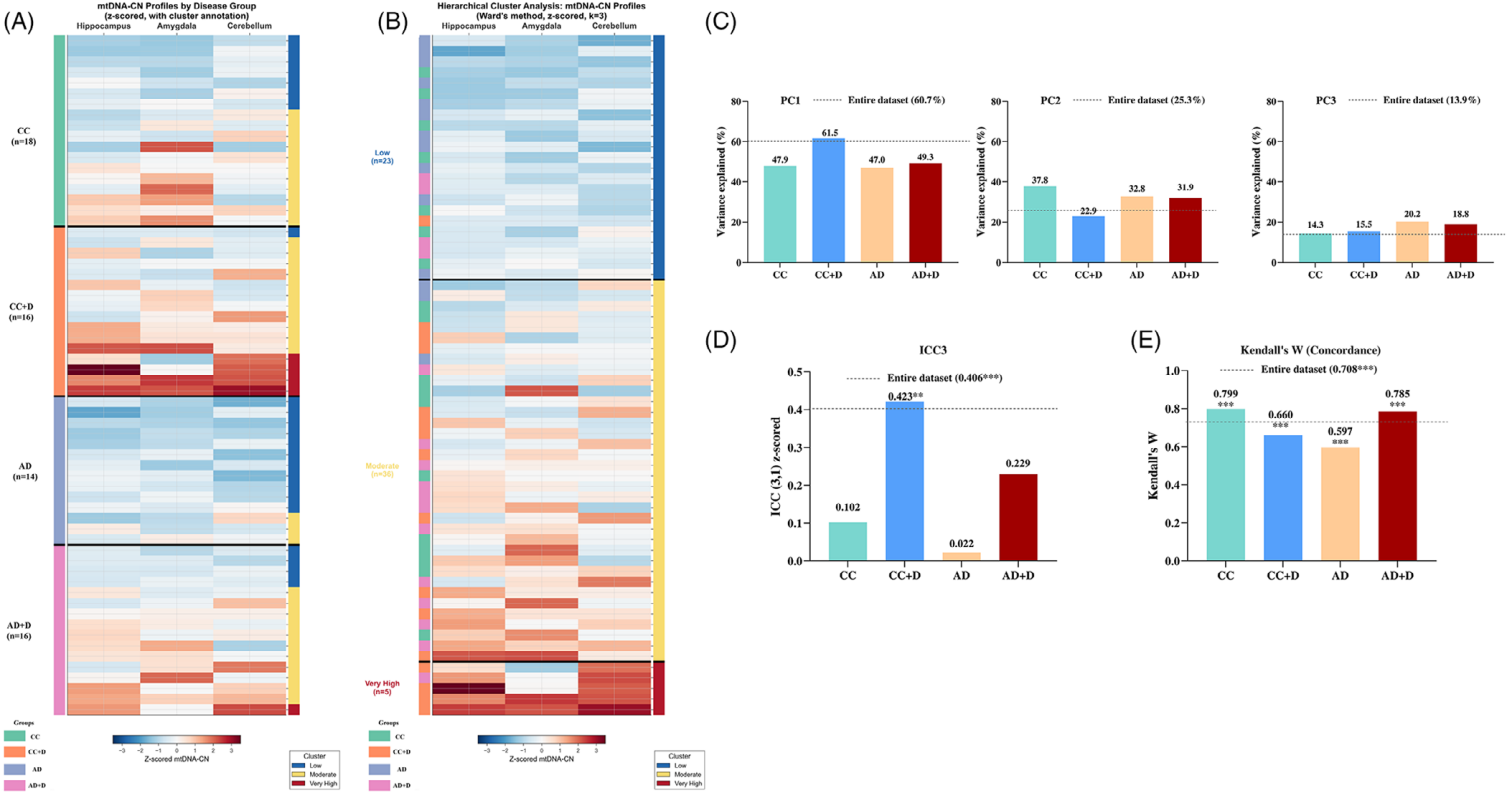
Regional distribution and intra‐individual patterns of mitochondrial DNA copy number (mtDNA‐CN) in hippocampus, amygdala, and cerebellum. Levels of mtDNA‐CN across the three brain regions in each individual were *z*‐scored and are shown as heatmaps. (A) Donors are ordered by disease group (CC, CC+D, AD, and AD+D). Within each group, donors are further ordered by hierarchical cluster assignment and mean *z*‐score. The left color bar indicates disease group, and the right color bar indicates cluster assignment (blue = low, yellow = moderate, red = very high). Case IDs are shown on the right, and black lines separate the disease groups. (B) Donors are ordered by hierarchical clustering using Ward's minimum variance method (*k* = 3), identifying three mtDNA‐CN profiles: low (*n* = 23), moderate (*n* = 36), and very high (*n* = 5). The low cluster was enriched for AD cases (48%), whereas the very high cluster was dominated by CC+D cases (80%). A chi‐square test of independence showed that the distribution of cases across the three mtDNA‐CN clusters differed significantly among the four disease groups (*χ* ^2^ = 24.2, *df* = 6, *p* = 0.0005). (C) The main patterns in the mtDNA‐CN dataset across the three brain regions were examined using principal component analysis (PCA). Principal component 1 (PC1) reflected the dominant shared mtDNA‐CN pattern across hippocampus, amygdala, and cerebellum, whereas PC2 and PC3 captured smaller region‐specific variation. The percentage of total variance explained by PC1, PC2, and PC3 across the disease groups is shown. The *x*‐axis shows the disease groups, and the *y* axis shows the percentage of total variance explained by each component. Dashed lines indicate the corresponding variance explained by each component in the whole dataset. PC1 explained 60.7% of the total variance in the whole dataset. Statistical comparison of donor‐level PC scores showed that PC1 differed significantly between groups (Kruskal–Wallis *H* = 26.13, *p* < 0.0001), whereas PC2 and PC3 showed no significant group differences. (D) Intraclass correlation coefficient (ICC) was used to assess within‐individual concordance in mtDNA‐CN across hippocampus, amygdala, and cerebellum. The whole dataset showed moderate within‐individual concordance, but this was not uniform across groups. CC+D showed the strongest concordance and was the only group with a significant ICC (*p* = 0.003). (E) Kendall's *W* was used to assess the consistency of regional ranking in mtDNA‐CN across hippocampus, amygdala, and cerebellum in the whole dataset and within each disease group. Kendall's *W* was significant in the whole dataset (*W* = 0.708 *p *< 0.001) and remained significant in all four disease groups (*p* < 0.001).

PCA of the whole dataset (Figure 4C) showed that the first principal component (PC1) accounted for 60.7% of the total variance, with all three brain regions loading positively and with similar magnitude (Table S5). This indicates that a large part of the variation reflects a shared mtDNA‐CN pattern seen consistently across amygdala, hippocampus, and cerebellum, where all regions tend to change together. PC2 explained an additional 25.3% of the variance and primarily captures divergence between amygdala and cerebellum, whereas PC3 (13.9%) reflected variation that was more hippocampus specific (Figure 4C, Table S5). Taken together, these components demonstrate that the dataset contains both a strong common mtDNA‐CN pattern across regions and meaningful region‐specific differences.

We next assessed whether this whole dataset structure was retained when donors were stratified into the four groups (Figure 4C, Table S5B–C). PC1 scores differed significantly across the four disease groups (Kruskal–Wallis *H* = 26.13, *p* < 0.0001, Table S5B), whereas PC2 and PC3 did not (Table S5B). Because PC1 was the dominant component and the only PC that differed significantly across disease groups (Table S5B), group‐specific loading analysis was focused on PC1. In CC+D, PC1 explained 61.5% of the variance and all three regions loaded equally (Table S5C), indicating a robust shared pattern within this group. In contrast, PC1 explained a smaller proportion of variance in the remaining groups (47.0%–49.3%), suggesting more heterogeneous within‐group structure and weaker uniformity across regions (Figure 4C, Table S5C). In both CC and AD, hippocampus and amygdala loaded together on PC1, whereas cerebellum loaded in the opposite direction (Table S5C). CC+D donors had the highest mean PC1 scores (+1.02 ± 1.66, Table S5B), whereas AD donors had the lowest (−1.22 ± 0.57, Table S5B). These opposing shifts are consistent with diabetes being associated with elevated and AD with reduced mtDNA‐CN across all three regions.

Intraclass correlation (ICC) analysis of the full dataset showed moderate within‐individual consistency across the three brains regions (ICC3 = 0.406, *p* < 0.001, Figure 4D, Table S6A). When examined separately by disease group (Figure 4D, Table S6A), CC+D showed the strongest regional concordance (ICC3 = 0.423, *p* = 0.003) and was the only group with statistically significant within‐individual agreement. In contrast, AD showed virtually no concordance (ICC3 = 0.022, *p* = 0.425), indicating that mtDNA‐CN levels in one region did not correlate with other regions in this group. Both CC and AD+D showed only weak and non‐significant concordance (Figure 4D, Table S6A). These results indicate that the moderate ICC observed in the combined dataset does not reflect a uniform pattern across all disease groups.

In contrast, Kendall's *W*, which assesses the consistency of regional ranking rather than absolute agreement, was significant in the full dataset (*W* = 0.708, *p* < 0.001) and remained significant within each of the four groups (Figure 4E, Table S6B). This indicates that the relative ordering of regions was generally preserved across individuals, even when absolute concordance was weak.

## 4 DISCUSSION

Diabetes is a well‐established risk factor for AD, but whether it accelerates canonical AD pathology or contributes through distinct metabolic mechanisms remains unclear.^2^, ^5^ Diabetes and AD share overlapping biological processes, including inflammation, oxidative stress, impaired insulin signaling, and mitochondrial dysfunction, which may converge to influence neurodegeneration.^6^, ^7^, ^29^ In this study, we examined mtDNA‐CN across three regions of the human brain, amygdala, and hippocampus, early and vulnerable targets of AD pathology,^29^, ^30^ and cerebellum, thought to be relatively resistant,^31^ to determine whether AD is associated with loss of mitochondrial content and if diabetes modifies regional mtDNA patterns in AD.

The central finding of this study is that mtDNA‐CN shows opposing directional changes in AD and diabetes, which can influence pooled analyses and obscure AD‐associated differences (Figure 3, Table 2). In the full dataset, mtDNA‐CN appeared modestly reduced in AD across regions, but these differences did not withstand correction for multiple comparisons. After diabetic cases were removed, AD associated reductions became larger, reaching significance in the amygdala and cerebellum, with effect sizes of similar magnitude across all three regions. In contrast, diabetes was associated with significantly higher mtDNA‐CN across all three brain regions (Figure 3). This opposing pattern explains why AD‐associated reductions were attenuated when diabetic donors were included. These findings should not be interpreted as evidence that mtDNA‐CN is uniformly altered in all AD cases; however, they show that diabetes is a strong modifier of mtDNA‐CN in the brain and can obscure AD‐associated mtDNA changes in pooled analyses

These findings are consistent with our previous report, where in a smaller, independent cohort, mtDNA‐CN measured in frontal cortex, parietal cortex, and cerebellum showed similar directional trends. Diabetes was associated with higher mtDNA‐CN across regions, whereas AD was associated with lower mtDNA‐CN in parietal cortex only.^12^ The present study extends these observations by showing comparable diabetes‐ and AD‐associated patterns in additional brain regions.

The second key finding in our study is that although mtDNA levels vary between brain regions, as shown in both our earlier^12^ and current study, across the different brain regions, similar trends of diabetes‐ or AD‐associated mtDNA‐CN changes are seen, as all regions tended to change together in the presence of diabetes. Only CC+D donors exhibited significant within‐individual concordance across regions, whereas AD donors showed no cross‐regional agreement at all. Nevertheless, Kendall's *W* indicated that the relative ordering of regions was preserved across all groups, even when absolute values diverged. Generally, the highest levels were found in amygdala (197.7–1098 copies/cell), slightly lower in hippocampus (63.2–1108 copies/cell), and significantly lower in cerebellum (38.0–376.2 copies/cell).

Our results support diabetes as an effect modifier of mtDNA‐CN patterns in AD brain rather than a background comorbidity. Pooled analyses that do not account for diabetes status may underestimate dementia‐associated reductions in mtDNA‐CN, with the observed effect depending on the metabolic composition of the cohort. This may help explain inconsistent mtDNA‐CN findings across human AD brain studies, where differences are often modest and not consistently detected (Table S7). Diabetes status is not systematically incorporated as a covariate in many large‐scale analyses, leaving open the possibility that metabolic heterogeneity contributes to inconsistency in mtDNA‐CN estimates.^23^ Although the present study used a matched design to balance AD and control participants with and without diabetes, diabetes is more prevalent in AD in the general population,^27^ which may amplify confounding in unstratified cohorts. Replication in larger independent cohorts will be required to confirm the robustness and biological significance of these findings.

Neither age nor sex was associated with mtDNA‐CN in this study. This aligns with large‐scale sequencing studies suggesting that mtDNA‐CN largely appears uninfluenced by age and sex in post‐mortem brain tissue. For instance, Rice et al. (2014) reported no correlation between mtDNA‐CN and age in either AD or control cohorts.^21^ Similarly, analyses from Klein et al. (2021) found no effect of age or sex on mtDNA‐CN after accounting for regional neuropathology across multiple brain regions, including the dorsolateral prefrontal cortex, posterior cingulate cortex, and cerebellum.^23^ Wei et al. (2017) further highlighted disease‐specific patterns, reporting no age association in AD but an age‐dependent increase in Creutzfeldt–Jakob disease.^24^ The present cohort was not sufficiently powered to test sex‐specific effects after stratification by diagnosis, diabetes status, and brain region. Larger studies are required to test whether AD‐diabetes associations with mtDNA‐CN differ between males and females.

A key question is whether the higher mtDNA‐CN seen in diabetic brain reflects improved mitochondrial capacity or is a maladaptive response to metabolic stress. Although mtDNA‐CN quantifies mitochondrial genome abundance, increased mtDNA‐CN does not necessarily indicate increased mitochondrial mass or improved mitochondrial function. In our previous in vitro studies using human renal mesangial and proximal tubular cells, sustained high‐glucose exposure progressively increased mtDNA‐CN without a corresponding increase in transcription or translation of mtDNA‐encoded genes. This preceded impaired mitochondrial respiration, reduced metabolic flexibility, and cellular damage, consistent with a compensatory response to glucotoxic stress rather than improved bioenergetic capacity.^7^, ^19^ We reported a similar sequence in a mouse model of nonalcoholic fatty liver disease (NAFLD), where an early maladaptive increase in hepatic mtDNA preceded reduction in oxidative phosphorylation (OXPHOS) proteins and liver damage.^35^ In patients with severe diabetic retinopathy and nephropathy, cell‐free mtDNA was increased whereas cellular mtDNA‐CN was reduced, alongside increased inflammation, mtDNA damage, and a disconnect between mtDNA content and mtDNA transcription.^7^, ^14^, ^36^ Thus, the higher mtDNA‐CN in diabetic brain may reflect metabolic stress or altered mitochondrial signaling rather than functional adaptation, although further studies are needed to define its bioenergetic impact.

In contrast to our finding of higher mtDNA‐CN in diabetic brain, peripheral studies generally suggest that lower circulating mtDNA‐CN is associated with increased risk of metabolic diseases, including chronic kidney disease,^18^, ^37^ cardiovascular disease,^15^, ^16^ and obesity.^13^ However, few studies have examined the long‐term impact of metabolic disease on mtDNA‐CN in target organs and tissues. It therefore remains unclear whether the brain pattern observed here is distinct from the periphery and vascular system, or whether other organs affected by diabetes show similar tissue‐specific responses.

Our results raise the possibility that diabetic and non‐diabetic AD are not biologically equivalent with respect to mitochondrial dysfunction. Because mtDNA‐CN represents only one mitochondrial readout, other mitochondrial and metabolic markers may also differ according to diabetes status. This has implications for biomarker interpretation and therapeutic development, as failure to account for diabetes may obscure biologically distinct processes within clinically defined AD cohorts and influence the apparent efficacy of metabolic or mitochondria‐targeted interventions. Future studies should assess whether diabetic and non‐diabetic AD differ across broader mitochondrial and metabolic profiles, and whether these differences are associated with treatment response.

To account for potential impact of antidiabetic treatment on mitochondrial function, we examined medication records and found no associations between mtDNA‐CN and metformin or other medications for our cohort (Table S8). However, the small number of treated individuals and incomplete lifetime treatment histories limit the interpretation. Larger cohorts with detailed data on medication class, dose, duration, and timing relative to death are required to distinguish diabetes‐associated effects from treatment‐associated effects.

In this context, trials of metabolic therapies, including glucagon‐like peptide‐1 receptor agonists such as semaglutide, may be informative if outcomes are examined with reference to diabetes status. For example, EVOKE/EVOKE+ could be examined for differential outcomes in AD patients with and without type 2 diabetes, given the opposing effects of AD and diabetes on mtDNA‐CN described here.^38^, ^39^ Public reporting in late 2025 indicated that EVOKE/EVOKE+ did not meet primary efficacy endpoints for slowing clinical progression overall, and early subgroup analyses suggested a similar lack of efficacy in patients with type 2 diabetes.^40^ Even when overall efficacy is neutral, such large datasets provide an opportunity to test whether metabolic status modifies biomarker trajectories or treatment response.^40^

A unique strength of the present study is the measurement of mtDNA‐CN in the three brain regions from the same 64 donors, enabling assessment of whether mtDNA‐CN levels are coordinated within individuals rather than evaluating each region in isolation. We have shown that diabetes is associated with a higher multiregional mtDNA‐CN profile across the brain, whereas AD, especially in the absence of diabetes, is associated with lower and more regionally variable mtDNA. These contrasting trends show that metabolic status influences AD and has potential therapeutic implications. The distinct multiregional pattern seen in AD+D versus AD also suggests that metabolic status may influence mitochondrial therapeutic responsiveness.

In summary, our findings show that AD is associated with cross‐regional reduced mtDNA‐CN, indicating a systemic loss of mitochondrial content, and supporting the broader view that impaired mitochondrial function contributes to AD pathology, potentially through reduced bioenergetic capacity. This effect was most evident in non‐diabetic AD and was masked when diabetic AD cases were included. These findings support treating diabetes as an effect modifier rather than an optional covariate in AD studies. Wherever possible, diabetes‐stratified analyses and formal diabetes‐AD interaction testing alongside sensitivity analyses incorporating broader metabolic measures (e.g., obesity measures, glycemic control, and medication exposure) could provide valuable information. This would strengthen causal inference, improve biomarker interpretation, and prevent diabetes‑related biology from being misattributed to AD.

## CONFLICT OF INTEREST STATEMENT

Any author disclosures are available in the supporting information.

## CONSENT STATEMENT

All donors had provided written informed consent for the use of postmortem brain for research purposes to the London Neurodegenerative Disease Brain Bank.

## Supporting information



Supporting Information

Supporting Information
