# Supplementary material for: Why diabetes matters in dementia studies: Failure to account for diabetes status masks regional mitochondrial DNA copy number changes in human hippocampus, amygdala, and cerebellum in Alzheimer's disease
Source: Alzheimers Dement. 2026 Oct 20;22(10):e71870. doi: 10.1002/alz.71870 (PMC13681739; doi:10.1002/alz.71870)
Supplement: Supplementary file 2 [file ALZ-22-e71870-s001.docx]

**Table S1: Oligonucleotide primers**

| **Gene ID**  **(accession number)** | **Oligonucleotide sequence (5’-3’)** | **Product size (bp)** |
| --- | --- | --- |
| hMito  (NC_012920.1) | F: CGCTTTCCACACAGACATCA | 127 |
|  | R: TGGTTAGGCTGGTGTTAGGG |  |
| hB2M (NC_000015.10) | F: TGTTCCTGCTGGGTAGCTCT | 189 |
|  | R: CCTCCATGATGCTGCTTACA |  |

**Abbreviations:** F: forward primer; R: reverse primer; hMito: human mitochondrial DNA; hB2M: human beta-2-microglobulin.

**Table S2: Demographic and clinical characteristics of post-mortem human brain samples and their average mitochondrial DNA copy number across three distinct regions.**

| **BBN ID** | **Sex** | **Age (years)** | **Post-mortem delay (hours)** | **Diabetes status**  **(Yes/No)** | **Cognitive status** | **MtDNA-CN (copies/cell)** | | |
| --- | --- | --- | --- | --- | --- | --- | --- | --- |
|  |  |  |  |  |  | **Hippocampus** | **Amygdala** | **Cerebellum** |
| BBN_22594 | Female | 77 | 21 | No | CC | 195.0 | 199.3 | 97.6 |
| BBN_23398 | Male | 83 | 31 | No | CC | 361.2 | 512.6 | 114.4 |
| BBN002.35137 | Female | 80 | 57 | No | CC | 699.8 | 407.7 | NA |
| BBN002.29089 | Male | 81 | 10 | No | CC | 347.9 | 987.7 | 133.1 |
| BBN002.28414 | Female | 68 | 59 | No | CC | 591.0 | 897.8 | 155.5 |
| BBN002.28350 | Male | 74 | 72 | No | CC | 175.2 | 209.0 | 150.1 |
| BBN002.26151 | Male | 82 | 26 | No | CC | 313.7 | 217.5 | 145.0 |
| BBN002.26311 | Male | 80 | 34 | No | CC | 267.2 | 601.5 | 126.7 |
| BBN_24666 | Female | 74 | 66 | No | CC | 600.7 | 850.0 | 89.6 |
| BBN_21801 | Male | 68 | 60 | No | CC | 196.0 | 1027.3 | 73.8 |
| BBN_24370 | Female | 68 | 53 | No | CC | 545.2 | 560.0 | 213.2 |
| BBN_24243 | Female | 69 | 48 | No | CC | 418.0 | 333.2 | 84.1 |
| BBN_22991 | Female | 73 | 27 | No | CC | 317.3 | 491.5 | 195.8 |
| BBN_24297 | Female | 83 | 39 | No | CC | 513.4 | 505.4 | 161.2 |
| BBN_20040 | Female | 80 | 22 | No | CC | 737.1 | 524.1 | NA |
| BBN_14408 | Male | 90 | 70 | No | CC | 367.9 | 335.0 | 216.5 |
| BBN_10993 | Female | 85 | 51 | No | CC | 235.3 | 275.7 | 137.0 |
| BBN_10214 | Male | 67 | 26 | No | CC | 311.5 | 237.0 | 170.3 |
| BBN_10209 | Male | 63 | 51 | No | CC | 435.9 | 776.5 | 151.0 |
| BBN002.36235 | Male | 84 | 32 | No | CC | 230.1 | 386.1 | 184.8 |
| BBN002.29056 | Female | 69 | 73 | No | AD | 386.9 | 382.7 | 164.3 |
| BBN002.28762 | Male | 86 | 70 | No | AD | 310.2 | 243.0 | 38.0 |
| BBN002.29910 | Female | 69 | 88 | No | AD | 316.3 | 569.7 | 150.8 |
| BBN002.30840 | Male | 72 | 61 | No | AD | 152.4 | 429.7 | NA |
| BBN002.30846 | Female | 100 | 18 | No | AD | 732.7 | 318.7 | NA |
| BBN002.31486 | Female | 89 | 9 | No | AD | 480.8 | 303.8 | 115.5 |
| BBN002.32545 | Female | 75 | 35 | No | AD | 194.6 | 285.6 | 142.7 |
| BBN002.32818 | Male | 74 | 31 | No | AD | 176.7 | 290.8 | 212.1 |
| BBN002.35184 | Male | 70 | 56 | No | AD | 282.2 | 469.9 | 90.4 |
| BBN002.35898 | Female | 73 | 50 | No | AD | 388.3 | 417.8 | 47.7 |
| BBN002.35951 | Male | 75 | 36 | No | AD | 482.8 | 299.4 | NA |
| BBN002.28120 | Female | 73 | 72 | No | AD | 142.5 | 384.0 | NA |
| BBN002.36128 | Male | 77 | 67 | No | AD | 236.7 | 251.5 | 63.2 |
| BBN_24558 | Female | 84 | 27 | No | AD | 503.0 | 614.3 | NA |
| BBN_23396 | Male | 83 | 71 | No | AD | 356.3 | 204.8 | 119.6 |
| BBN_16191 | Male | 88 | 33 | No | AD | 370.8 | 354.5 | 86.1 |
| BBN_10192 | Female | 84 | 27 | No | AD | 171.0 | 300.9 | 84.5 |
| BBN_9985 | Female | 88 | 67 | No | AD | 304.8 | 446.5 | 55.6 |
| BBN_4570 | Female | 86 | 71 | No | AD | 63.2 | 218.9 | 135.7 |
| BBN002.32557 | Female | 77 | 50 | No | AD | 341.7 | 430.4 | NA |
| BBN_16694 | Male | 75 | 85 | Yes | CC+D | 1107.6 | 516.8 | 311.2 |
| BBN002.26310 | Female | 93 | 72 | Yes | CC+D | 589.2 | 246.3 | 128.8 |
| BBN002.30139 | Female | 47 | 27 | Yes | CC+D | 846.9 | 1044.6 | 181.5 |
| BBN002.32840 | Female | 88 | 15 | Yes | CC+D | 326.0 | 311.9 | 249.3 |
| BBN002.33675 | Male | 90 | 12 | Yes | CC+D | 676.2 | 589.2 | 177.5 |
| BBN002.33764 | Male | 73 | 49 | Yes | CC+D | 272.1 | 1037.1 | NA |
| BBN002.35541 | Male | 75 | 40 | Yes | CC+D | 894.5 | 1036.6 | 376.2 |
| BBN002.36344 | Male | 79 | 37 | Yes | CC+D | 365.3 | 683.4 | 154.4 |
| BBN_9958 | Female | 84 | 53 | Yes | CC+D | 274.3 | 599.0 | 133.4 |
| BBN_4194 | Male | 97 | 44 | Yes | CC+D | 354.6 | 497.3 | 154.7 |
| BBN_16192 | Female | 81 | 30 | Yes | CC+D | 279.4 | 544.1 | 266.0 |
| BBN002.26316 | Female | 95 | 53 | Yes | CC+D | 673.2 | 618.9 | 194.5 |
| BBN002.32584 | Female | 98 | 25 | Yes | CC+D | 329.3 | 393.6 | 111.4 |
| BBN002.32590 | Female | 81 | 27 | Yes | CC+D | 391.4 | 706.9 | 117.1 |
| BBN_16307 | Female | 55 | 12 | Yes | CC+D | 612.1 | 444.2 | 107.4 |
| BBN002.35480 | Female | 79 | 56 | Yes | CC+D | 750.1 | 1097.5 | 317.9 |
| BBN_16429 | Male | 68 | 53 | Yes | CC+D | 275.0 | 197.7 | 452.0 |
| BBN002.37301 | Male | 62 | 75 | Yes | CC+D | 535.8 | 213.7 | 298.4 |
| BBN_23793 | Male | 84 | 67 | Yes | AD+D | 436.1 | 553.1 | 175.1 |
| BBN002.28760 | Male | 76 | 55 | Yes | AD+D | 535.6 | 578.7 | 141.6 |
| BBN002.33690 | Male | 84 | 40 | Yes | AD+D | 338.8 | 274.4 | 124.4 |
| BBN002.30168 | Female | 68 | 43 | Yes | AD+D | 686.4 | 703.0 | 243.5 |
| BBN_9851 | Female | 82 | 12 | Yes | AD+D | 311.0 | 368.5 | 142.4 |
| BBN_9845 | Male | 81 | 74 | Yes | AD+D | 224.6 | 508.0 | NA |
| BBN002.30072 | Female | 82 | 66 | Yes | AD+D | 511.7 | 640.1 | 154.7 |
| BBN002.33768 | Female | 92 | 27 | Yes | AD+D | 286.9 | 388.1 | NA |
| BBN002.36940 | Male | 81 | 40 | Yes | AD+D | 501.9 | 399.0 | 145.5 |
| BBN_4195 | Male | 83 | 77 | Yes | AD+D | 309.2 | 452.0 | 129.0 |
| BBN_9884 | Male | 74 | 51 | Yes | AD+D | 305.0 | 462.5 | 235.2 |
| BBN_9927 | Female | 90 | 35 | Yes | AD+D | 511.0 | 771.8 | 704.6 |
| BBN_4241 | Female | 94 | 47 | Yes | AD+D | 546.8 | 819.8 | 80.0 |
| BBN_11075 | Male | 78 | 24 | Yes | AD+D | 710.1 | 506.3 | 218.8 |
| BBN_16202 | Male | 67 | 22 | Yes | AD+D | 718.7 | 506.3 | 323.4 |
| BBN_19996 | Female | 75 | 12 | Yes | AD+D | 562.1 | 465.4 | 179.3 |
| BBN002.26678 | Male | 86 | 38 | Yes | AD+D | 333.9 | 399.5 | 90.0 |
| BBN002.32884 | Female | 82 | 98 | Yes | AD+D | 307.7 | 636.9 | 289.5 |
| BBN002.32575 | Female | 89 | 59 | Yes | AD+D | 440.8 | 985.6 | 148.3 |

**Abbreviations:** AD: Alzheimer's disease; AD+D: Alzheimer's disease with diabetes; CC: Cognitive controls; CC+D: Cognitive controls with diabetes; BBN UK: Brain Banks Network; NA: Not available.

**Table S3A: Raw data showing mtDNA and nuclear DNA copy numbers obtained by real-time qPCR from human hippocampus**

| **BBN ID** | **Copy number of mtDNA amplicons (hMitoF3-R3)** | | | **Average copy number of mtDNA amplicons**  **(Mt)** | **CoV** | **Copy number of nuclear DNA amplicons (hB2MF1-R1)** | | | **Average copy number of nDNA amplicons**  **(N)** | **CoV** | **MtDNA-CN (copies/cell** |
| --- | --- | --- | --- | --- | --- | --- | --- | --- | --- | --- | --- |
|  | **Replicate 1** | **Replicate 2** | **Replicate 3** |  |  | **Replicate 1** | **Replicate 2** | **Replicate 3** |  |  |  |
| BBN_22594 | 4245972 | 3933821 | 4365522 | 4181772 | 5.33 | 40038 | 42324 | 46319 | 42894 | 7.41 | 195.0 |
| BBN_23398 | 7985854 | 7146362 | 6713547 | 7281921 | 8.88 | 41452 | 39486 | 40038 | 40325 | 2.51 | 361.2 |
| BBN002.35137 | 22310417 | 22779910 | 21549333 | 22213220 | 2.80 | 66448 | 61139 | 62860 | 63482 | 4.27 | 699.8 |
| BBN002.29089 | 9303500 | 7660070 | 8441887 | 8468486 | 9.71 | 45365 | 50692 | 49994 | 48684 | 5.95 | 347.9 |
| BBN002.28414 | 10990081 | 10990081 | 11221353 | 11067172 | 1.21 | 38672 | 37354 | 36331 | 37452 | 3.13 | 591.0 |
| BBN002.28350 | 3150227 | 2760967 | 2858479 | 2923224 | 6.93 | 34849 | 32739 | 32513 | 33367 | 3.86 | 175.2 |
| BBN002.26151 | 7249267 | 6614023 | 6708001 | 6857097 | 5.00 | 41881 | 43688 | 45574 | 43714 | 4.22 | 313.7 |
| BBN002.26311 | NA | 6295346 | 6251092 | 6273219 | 0.50 | 50651 | 45574 | 44621 | 46949 | 6.90 | 267.2 |
| BBN_24666 | 16548171 | 17385856 | 15862354 | 16598794 | 4.60 | 57092 | 53586 | 55117 | 55265 | 3.18 | 600.7 |
| BBN_21801 | 4918008 | 4714188 | 4849107 | 4827101 | 2.15 | 47541 | 51009 | 49244 | 49265 | 3.52 | 196.0 |
| BBN_24370 | 14406502 | 13916933 | 13631214 | 13984883 | 2.80 | 48829 | 53090 | 51991 | 51303 | 4.31 | 545.2 |
| BBN_24243 | 11226519 | 10914157 | 10991423 | 11044033 | 1.47 | 54346 | 52466 | 51732 | 52848 | 2.55 | 418.0 |
| BBN_22991 | 6803315 | 7456740 | 6998025 | 7086027 | 4.73 | 47541 | 42475 | 43997 | 44671 | 5.82 | 317.3 |
| BBN_24297 | 12632855 | 13167991 | 11546822 | 12449222 | 6.64 | 49514 | 48489 | 47485 | 48496 | 2.09 | 513.4 |
| BBN_20040 | 18478498 | 16091873 | 17363595 | 17311322 | 6.90 | 47485 | 44597 | 48829 | 46970 | 4.60 | 737.1 |
| BBN_14408 | 11148714 | 11549349 | 10536404 | 11078156 | 4.60 | 63248 | 57678 | 59760 | 60229 | 4.67 | 367.9 |
| BBN_10993 | 6753053 | 6800905 | 6800905 | 6784954 | 0.41 | 57270 | 58088 | 57678 | 57679 | 0.71 | 235.3 |
| BBN_10214 | 8831385 | 8585442 | 8171407 | 8529411 | 3.91 | 52973 | 55669 | 55669 | 54770 | 2.84 | 311.5 |
| BBN_10209 | 12570601 | 12749381 | 13301125 | 12873702 | 2.96 | 58088 | 59337 | 59760 | 59062 | 1.47 | 435.9 |
| BBN002.36235 | 6611508 | 6204426 | 6658357 | 6491430 | 3.85 | 60613 | 55669 | 52973 | 56418 | 6.87 | 230.1 |
| BBN002.29056 | 9887660 | 10536404 | 10686255 | 10370106 | 4.09 | 54112 | 54112 | 52598 | 53607 | 1.63 | 386.9 |
| BBN002.28762 | 8831385 | 8346348 | 8646278 | 8608004 | 2.84 | 51127 | 58502 | 56866 | 55498 | 6.98 | 310.2 |
| BBN002.29910 | 8465051 | 8646278 | 8113912 | 8408414 | 3.22 | 54885 | 55275 | 49346 | 53169 | 6.24 | 316.3 |
| BBN002.30840 | 4452194 | 4483742 | 4612186 | 4516040 | 1.88 | 61917 | 55669 | 60185 | 59257 | 5.44 | 152.4 |
| BBN002.30846 | 17150783 | 20606968 | 21197287 | 19651680 | 11.12 | 50766 | 54885 | 55275 | 53642 | 4.66 | 732.7 |
| BBN002.31486 | 10761977 | 10462269 | 10099344 | 10441197 | 3.18 | 43742 | 43742 | 42821 | 43435 | 1.22 | 480.8 |
| BBN002.32545 | 3290021 | 2998566 | 3266629 | 3185072 | 5.08 | 30844 | 32627 | 34757 | 32743 | 5.98 | 194.6 |
| BBN002.32818 | 5822465 | 5578453 | 5460309 | 5620409 | 3.29 | 64958 | 63158 | 62716 | 63611 | 1.87 | 176.7 |
| BBN002.35184 | 7690635 | 7801173 | 7690635 | 7727481 | 0.83 | 54493 | 56047 | 53733 | 54758 | 2.15 | 282.2 |
| BBN002.35898 | 10831963 | 9663301 | 9943080 | 10146114 | 6.01 | 50796 | 53357 | 52612 | 52255 | 2.52 | 388.3 |
| BBN002.35951 | 13322137 | 11225390 | 13039993 | 12529173 | 9.08 | 53733 | 51878 | 50087 | 51899 | 3.51 | 482.8 |
| BBN002.28120 | 4667050 | 4376760 | 4700470 | 4581427 | 3.89 | 69687 | 61407 | 61840 | 64311 | 7.25 | 142.5 |
| BBN002.36128 | 7635955 | 7161000 | 7161000 | 7319318 | 3.75 | 60977 | 61407 | 63158 | 61848 | 1.87 | 236.7 |
| BBN_24558 | 12228908 | 12055631 | 12855224 | 12379921 | 3.40 | 51515 | 50440 | 45715 | 49223 | 6.27 | 503.0 |
| BBN_23396 | 9258324 | 10014281 | 9324622 | 9532409 | 4.39 | 51515 | 53733 | 55265 | 53504 | 3.52 | 356.3 |
| BBN_16191 | 11884810 | 10831963 | 10527173 | 11081315 | 6.43 | 63158 | 60127 | 56047 | 59777 | 5.97 | 370.8 |
| BBN_10192 | 4471460 | 4503479 | 4568208 | 4514383 | 1.09 | 50087 | 51878 | 56442 | 52802 | 6.21 | 171.0 |
| BBN_9985 | 6910021 | 7110085 | 6959503 | 6993203 | 1.49 | 45715 | 42913 | 49042 | 45890 | 6.69 | 304.8 |
| BBN_4570 | 2039743 | 2025240 | 1912871 | 1992618 | 3.48 | 63158 | 63158 | 62716 | 63010 | 0.41 | 63.2 |
| BBN002.32557 | 7315941 | 7110085 | 6910021 | 7112016 | 2.85 | 42613 | 40854 | 41432 | 41633 | 2.15 | 341.7 |
| BBN_16694 | 24931250 | 24758782 | 24587507 | 24759180 | 0.69 | 42324 | 43515 | 48289 | 44709 | 7.06 | 1107.6 |
| BBN002.26310 | 13915639 | 14208475 | 13628839 | 13917651 | 2.08 | 49648 | 47955 | 44123 | 47242 | 5.99 | 589.2 |
| BBN002.30139 | 11378232 | 11862150 | 10689117 | 11309833 | 5.21 | 24293 | 28497 | 27334 | 26708 | 8.13 | 846.9 |
| BBN002.32840 | 5566128 | 5413699 | 5451411 | 5477079 | 1.45 | 34608 | 34131 | 32065 | 33601 | 4.02 | 326.0 |
| BBN002.33675 | 12714847 | 12892605 | 12452794 | 12686748 | 1.74 | 34131 | 38405 | 40038 | 37525 | 8.13 | 676.2 |
| BBN002.33764 | 5924970 | 5883983 | 5722849 | 5843934 | 1.83 | 44740 | 41165 | 42952 | 42952 | 4.16 | 272.1 |
| BBN002.35541 | 13723776 | 13915639 | 13163912 | 13601109 | 2.87 | 31187 | 30544 | 29503 | 30411 | 2.79 | 894.5 |
| BBN002.36344 | 7404322 | 7198307 | 7249267 | 7283966 | 1.47 | 39867 | 38760 | 41006 | 39877 | 2.82 | 365.3 |
| BBN_9958 | 7404322 | 6295346 | 6339914 | 6679861 | 9.40 | 46876 | 48215 | 51009 | 48700 | 4.33 | 274.3 |
| BBN_4194 | 9545076 | 9149494 | 8957893 | 9217488 | 3.25 | 51009 | 51009 | 53965 | 51994 | 3.28 | 354.6 |
| BBN_16192 | 6899983 | 6998025 | 6384796 | 6760935 | 4.87 | 47876 | 45574 | 51732 | 48394 | 6.43 | 279.4 |
| BBN002.26316 | 14991940 | 13872568 | 15098075 | 14654194 | 4.63 | 44308 | 40718 | 45574 | 43533 | 5.79 | 673.2 |
| BBN002.32584 | 7616233 | 7456740 | 7670151 | 7581041 | 1.46 | 46547 | 43688 | 47876 | 46037 | 4.65 | 329.3 |
| BBN002.32590 | 9319001 | 9646825 | 9781161 | 9582329 | 2.48 | 47818 | 47818 | 51271 | 48969 | 4.07 | 391.4 |
| BBN_16307 | 13204160 | 14169288 | 12567958 | 13313802 | 6.06 | 45254 | 43078 | 42177 | 43503 | 3.64 | 612.1 |
| BBN002.35480 | 17974410 | 17605390 | 18865820 | 18148540 | 3.57 | 47485 | 49861 | 47818 | 48388 | 2.66 | 750.1 |
| BBN_16429 | 5904337 | 6415156 | 5625358 | 5981617 | 6.70 | 42472 | 42177 | 45858 | 43502 | 4.70 | 275.0 |
| BBN002.37301 | 12897648 | 12119466 | 11870649 | 12295921 | 4.36 | 44287 | 44909 | 48489 | 45895 | 4.94 | 535.8 |
| BBN_23793 | 10007092 | 9256374 | 9061591 | 9441685 | 5.29 | 42639 | 40872 | 46405 | 43305 | 6.53 | 436.1 |
| BBN002.28760 | 10895664 | 11369113 | 10516219 | 10926999 | 3.91 | 42639 | 39178 | 40585 | 40801 | 4.27 | 535.6 |
| BBN002.33690 | 7483011 | 7698243 | 7863721 | 7681658 | 2.49 | 47064 | 45114 | 43859 | 45346 | 3.56 | 338.8 |
| BBN002.30168 | 9455344 | 9190985 | 8997578 | 9214636 | 2.49 | 28124 | 26394 | 26024 | 26847 | 4.17 | 686.4 |
| BBN_9851 | 6872750 | 6586545 | 6921646 | 6793647 | 2.66 | 41453 | 43551 | 46079 | 43694 | 5.30 | 311.0 |
| BBN_9845 | 6516005 | 6338408 | 6165652 | 6340022 | 2.76 | 57362 | 57362 | 54632 | 56452 | 2.79 | 224.6 |
| BBN002.30072 | 9322228 | 8870906 | 8870906 | 9021347 | 2.89 | 34265 | 36254 | NA | 35259 | 3.99 | 511.7 |
| BBN002.33768 | 6357166 | 6223391 | 5635386 | 6071981 | 6.32 | 45433 | 42941 | 38630 | 42335 | 8.13 | 286.9 |
| BBN002.36940 | 8746018 | 8032755 | 8684234 | 8487669 | 4.66 | 34265 | 31707 | 35494 | 33822 | 5.71 | 501.9 |
| BBN_4195 | NA | 7430149 | 7808170 | 7619160 | 3.51 | 46733 | 48411 | 52687 | 49277 | 6.23 | 309.2 |
| BBN_9884 | 9268228 | 8590005 | 8891907 | 8916713 | 3.81 | 57362 | 56963 | 61072 | 58466 | 3.88 | 305.0 |
| BBN_9927 | 9796511 | 9796511 | 10368167 | 9987063 | 3.30 | 35494 | 37291 | 44482 | 39089 | 12.17 | 511.0 |
| BBN_4241 | 12388231 | 13002037 | 13002037 | 12797435 | 2.77 | 46222 | 44640 | 49556 | 46806 | 5.36 | 546.8 |
| BBN_11075 | 21829003 | 22132705 | 20942673 | 21634794 | 2.86 | 61072 | 61072 | 60648 | 60931 | 0.40 | 710.1 |
| BBN_16202 | 19544704 | 19680195 | 19680195 | 19635032 | 0.40 | 55398 | 55014 | 53502 | 54638 | 1.83 | 718.7 |
| BBN_19996 | 11168756 | 11967620 | 11015500 | 11383959 | 4.49 | 41637 | 40493 | 39381 | 40504 | 2.79 | 562.1 |
| BBN002.26678 | 9593967 | 8830689 | 9268228 | 9230961 | 4.15 | 55786 | 53502 | 56568 | 55285 | 2.88 | 333.9 |
| BBN002.32884 | 6208394 | 6471145 | 6745015 | 6474851 | 4.14 | 39381 | 42221 | 44640 | 42081 | 6.26 | 307.7 |
| BBN002.32575 | 10423217 | 10568234 | 10069324 | 10353592 | 2.48 | 46871 | 47528 | 46545 | 46981 | 1.07 | 440.8 |

**Abbreviations:** BBN UK: Brain Banks Network. The average copy numbers of mtDNA amplicons (Mt) and nDNA amplicons (N) for each sample were calculated as the mean of three technical replicates. mtDNA-CN was calculated as (Mt/N) × 2. CoV denotes the coefficient of variation, calculated as percentage of (standard deviation/mean) from the three technical replicates per sample; values <20% were considered acceptable.

**Table S3B. Raw data showing mtDNA and nuclear DNA copy numbers obtained by real-time qPCR from human amygdala**

| **BBN ID** | **Copy number of mtDNA amplicons (hMitoF3-R3)** | | | **Average copy number of mtDNA amplicons**  **(Mt)** | **CoV** | **Copy number of nuclear DNA amplicons (hB2MF1-R1)** | | | **Average copy number of nDNA amplicons**  **(N)** | **CoV** |  |
| --- | --- | --- | --- | --- | --- | --- | --- | --- | --- | --- | --- |
|  | **Replicate 1** | **Replicate 2** | **Replicate 3** |  |  | **Replicate 1** | **Replicate 2** | **Replicate 3** |  |  | **MtDNA-CN (copies/cell)** |
| BBN_22594 | 6241248 | 6241248 | 6283651 | 6255382 | 0.39 | 63212 | 64564 | 60591 | 62789 | 3.22 | 199.3 |
| BBN_23398 | 13876151 | 12536005 | 15050707 | 13820954 | 9.10 | 52988 | 51514 | 57265 | 53922 | 5.54 | 512.6 |
| BBN002.35137 | 12283926 | 13144490 | 14065343 | 13164586 | 6.77 | 70767 | 59742 | 63212 | 64574 | 8.73 | 407.7 |
| BBN002.29089 | 24673167 | 26046547 | 30230451 | 26983389 | 10.73 | 54122 | 52988 | 56863 | 54658 | 3.64 | 987.4 |
| BBN002.28414 | 30642623 | 31271443 | 32568058 | 31494041 | 3.12 | 67356 | 69285 | 73828 | 70156 | 4.74 | 897.8 |
| BBN002.28350 | 6633418 | 6412599 | 6861841 | 6635952 | 3.39 | 63659 | 62767 | 64110 | 63512 | 1.08 | 209.0 |
| BBN002.26151 | 5638474 | 5450775 | 5487808 | 5525686 | 1.80 | 49728 | 53364 | 49378 | 50823 | 4.34 | 217.4 |
| BBN002.26311 | 15463918 | 15050707 | 16105102 | 15539909 | 3.42 | 51514 | 49378 | 54122 | 51671 | 4.60 | 601.5 |
| BBN_24666 | 23530995 | 22902225 | 25522792 | 23985337 | 5.70 | 56463 | 52246 | 60591 | 56433 | 7.39 | 850.0 |
| BBN_21801 | 13782511 | 15255913 | 15781254 | 14939893 | 6.94 | 29086 | 28881 | 29292 | 29086 | 0.71 | 1027.3 |
| BBN_24370 | 10025763 | 10162458 | 10655734 | 10281318 | 3.22 | 35691 | 37235 | 37235 | 36720 | 2.43 | 560.0 |
| BBN_24243 | 7910327 | 7910327 | 8935653 | 8252102 | 7.17 | 46668 | 51151 | 50792 | 49537 | 5.03 | 333.2 |
| BBN_22991 | 11955687 | 12536005 | 11022666 | 11838119 | 6.45 | 46014 | 46998 | 51514 | 48175 | 6.09 | 491.5 |
| BBN_24297 | 14257115 | 16214521 | 17947915 | 16139850 | 11.44 | 60165 | 66412 | 65021 | 63866 | 5.14 | 505.4 |
| BBN_20040 | 15568981 | 14648538 | 12706925 | 14308148 | 10.21 | 52616 | 61452 | 49728 | 54599 | 11.19 | 524.1 |
| BBN_14408 | 8815460 | 9958107 | 9433037 | 9402201 | 6.08 | 52246 | 58079 | 58079 | 56135 | 6.00 | 335.0 |
| BBN_10993 | 4924344 | 5952328 | 5992768 | 5623147 | 10.77 | 37235 | 42278 | 42879 | 40797 | 7.60 | 275.7 |
| BBN_10214 | 4448756 | 5676782 | 4924344 | 5016627 | 12.34 | 39397 | 42879 | 44733 | 42336 | 6.40 | 237.0 |
| BBN_10209 | 18316225 | 19599390 | 21258393 | 19724669 | 7.48 | 51514 | 51514 | 49378 | 50802 | 2.43 | 776.5 |
| BBN002.36235 | 15568981 | 16324684 | 18819089 | 16904252 | 10.06 | 88072 | 92531 | 82071 | 87558 | 5.99 | 386.1 |
| BBN002.29056 | 14065343 | 13970427 | 15050707 | 14362159 | 4.17 | 75407 | 73828 | 75941 | 75059 | 1.46 | 382.7 |
| BBN002.28762 | 7751263 | 8018179 | 8238315 | 8002586 | 3.05 | 65021 | 70269 | 62326 | 65872 | 6.13 | 243.0 |
| BBN002.29910 | 15359564 | 15781254 | 17001559 | 16047459 | 5.31 | 56066 | 57265 | 55671 | 56334 | 1.47 | 569.7 |
| BBN002.30840 | 12536005 | 12201031 | 12880175 | 12539070 | 2.71 | 63212 | 52988 | 58905 | 58368 | 8.79 | 429.7 |
| BBN002.30846 | 7803926 | 8815460 | 7698955 | 8106114 | 7.61 | 52616 | 52988 | 46998 | 50867 | 6.60 | 318.7 |
| BBN002.31486 | 9180977 | 9180977 | 8294287 | 8885414 | 5.76 | 63212 | 53364 | 58905 | 58493 | 8.44 | 303.8 |
| BBN002.32545 | 7293006 | 7493232 | 8072655 | 7619631 | 5.31 | 53364 | 53364 | 53364 | 53364 | 0.00 | 285.6 |
| BBN002.32818 | 6369325 | 6241248 | 6678486 | 6429686 | 3.50 | 43796 | 45369 | 43488 | 44218 | 2.28 | 290.8 |
| BBN002.35184 | 11172953 | 11479702 | 12967685 | 11873446 | 8.09 | 46340 | 48004 | 57265 | 50536 | 11.65 | 469.9 |
| BBN002.35898 | 13414229 | 13144490 | 14353979 | 13637566 | 4.66 | 60591 | 69775 | 65482 | 65283 | 7.04 | 417.8 |
| BBN002.35951 | 6241248 | 5487808 | 5992768 | 5907275 | 6.50 | 41101 | 36198 | 41101 | 39467 | 7.17 | 299.4 |
| BBN002.28120 | 6074476 | 6723860 | 7442666 | 6747000 | 10.14 | 33025 | 35944 | 36455 | 35141 | 5.26 | 384.0 |
| BBN002.36128 | 4359298 | 4242814 | 4073897 | 4225336 | 3.40 | 30344 | 36973 | 33495 | 33604 | 9.87 | 251.5 |
| BBN_24558 | 13233795 | 13233795 | 11875007 | 12780866 | 6.14 | 37498 | 45050 | 42278 | 41609 | 9.18 | 614.3 |
| BBN_23396 | 6500030 | 7098129 | 7146355 | 6914838 | 5.21 | 67833 | 65945 | 68798 | 67525 | 2.15 | 204.8 |
| BBN_16191 | 11097555 | 12283926 | 12036915 | 11806132 | 5.30 | 58905 | 75407 | 65482 | 66598 | 12.47 | 354.5 |
| BBN_10192 | 9433037 | 8875353 | 8407374 | 8905254 | 5.77 | 60591 | 59742 | 57265 | 59199 | 2.92 | 300.9 |
| BBN_9985 | 13597123 | 13970427 | 13323707 | 13630419 | 2.38 | 58491 | 61452 | 63212 | 61051 | 3.91 | 446.5 |
| BBN_4570 | 6815535 | 7002653 | 7002653 | 6940280 | 1.56 | 56863 | 65021 | 68314 | 63399 | 9.30 | 218.9 |
| BBN002.32557 | 12451409 | 11325289 | 11794872 | 11857190 | 4.77 | 49378 | 61020 | 54891 | 55096 | 10.57 | 430.4 |
| BBN_16694 | 14065343 | 14257115 | 13970427 | 14097628 | 1.04 | 57265 | 51514 | 54891 | 54557 | 5.30 | 516.8 |
| BBN002.26310 | 6588654 | 6544192 | 6456166 | 6529671 | 1.03 | 46014 | 58905 | 54122 | 53013 | 12.29 | 246.3 |
| BBN002.30139 | 21694638 | 20690348 | 22594168 | 21659718 | 4.40 | 41392 | 35691 | 47331 | 41471 | 14.03 | 1044.6 |
| BBN002.32840 | 8579902 | 9306154 | 8464494 | 8783517 | 5.19 | 61020 | 56066 | 51878 | 56321 | 8.13 | 311.9 |
| BBN002.33675 | 13597123 | 13055788 | 11557696 | 12736869 | 8.29 | 44733 | 40240 | 44733 | 43235 | 6.00 | 589.2 |
| BBN002.33764 | 18316225 | 19599390 | 17233364 | 18382993 | 6.44 | 31880 | 37235 | 37235 | 35450 | 8.72 | 1037.1 |
| BBN002.35541 | 16105102 | 17350449 | 20001590 | 17819047 | 11.17 | 35191 | 34454 | 33495 | 34380 | 2.47 | 1036.6 |
| BBN002.36344 | 13970427 | 14748061 | 13233795 | 13984094 | 5.41 | 40525 | 42577 | 39676 | 40926 | 3.64 | 683.3 |
| BBN_9958 | 14353979 | 14353979 | 12118695 | 13608884 | 9.48 | 42278 | 48344 | 45690 | 45437 | 6.69 | 599.0 |
| BBN_4194 | 18819089 | 19866614 | 20412044 | 19699249 | 4.11 | 79226 | 80352 | 78115 | 79231 | 1.41 | 497.3 |
| BBN_16192 | 16324684 | 15674758 | 16772872 | 16257438 | 3.40 | 60165 | 61452 | 57671 | 59763 | 3.22 | 544.1 |
| BBN002.26316 | 14949141 | 16214521 | 16435596 | 15866419 | 5.05 | 42879 | 55671 | 55280 | 51277 | 14.19 | 618.9 |
| BBN002.32584 | 9824161 | 10948283 | 11636220 | 10802888 | 8.47 | 55671 | 54891 | 54122 | 54895 | 1.41 | 393.6 |
| BBN002.32590 | 17826798 | 17468329 | 17706498 | 17667208 | 1.03 | 48004 | 51878 | 50080 | 49987 | 3.88 | 706.9 |
| BBN_16307 | 8350639 | 8350639 | 9369380 | 8690219 | 6.77 | 41980 | 39957 | 35440 | 39126 | 8.56 | 444.2 |
| BBN002.35480 | 23214481 | 24673167 | 23214481 | 23700710 | 3.55 | 44106 | 43182 | 42278 | 43189 | 2.12 | 1097.5 |
| BBN_16429 | 7392441 | 7392441 | 7442666 | 7409183 | 0.39 | 71773 | 73309 | 79787 | 74956 | 5.67 | 197.7 |
| BBN002.37301 | 5952328 | 5600424 | 5198448 | 5583733 | 6.76 | 54122 | 50792 | 51878 | 52264 | 3.25 | 213.7 |
| BBN_23793 | 13782511 | 15781254 | 14848261 | 14804009 | 6.76 | 44418 | 57265 | 58905 | 53529 | 14.82 | 553.1 |
| BBN002.28760 | 15359564 | 14648538 | 15996421 | 15334841 | 4.40 | 51878 | 53364 | 53741 | 52994 | 1.86 | 578.7 |
| BBN002.33690 | 8935653 | NA | 9119022 | 9027337 | 1.44 | 67356 | 65945 | 64110 | 65804 | 2.47 | 274.4 |
| BBN002.30168 | 12367384 | 11875007 | 12967685 | 12403359 | 4.41 | 35691 | 36198 | 33971 | 35287 | 3.31 | 703.0 |
| BBN_9851 | 12201031 | 9306154 | 10231503 | 10579562 | 13.97 | 55671 | 57671 | 58905 | 57416 | 2.84 | 368.5 |
| BBN_9845 | 15050707 | 17117069 | 15674758 | 15947511 | 6.65 | 60591 | 63659 | 64110 | 62787 | 3.05 | 508.0 |
| BBN002.30072 | 11022666 | 11955687 | 12451409 | 11809921 | 6.14 | 34698 | 40812 | 35191 | 36900 | 9.21 | 640.1 |
| BBN002.33768 | 12283926 | 13597123 | 14451501 | 13444183 | 8.12 | 68798 | 70269 | 68798 | 69288 | 1.23 | 388.1 |
| BBN002.36940 | 10801018 | 10301016 | 10583826 | 10561954 | 2.37 | 49031 | 55671 | 54122 | 52941 | 6.56 | 399.0 |
| BBN_4195 | 12201031 | 11875007 | 10948283 | 11674774 | 5.57 | 50080 | 51151 | 53741 | 51658 | 3.64 | 452.0 |
| BBN_9884 | 16772872 | 15255913 | 14949141 | 15659309 | 6.24 | 71268 | 65945 | 65945 | 67720 | 4.54 | 462.5 |
| BBN_9927 | 18192623 | 16886828 | 17350449 | 17476633 | 3.79 | 43182 | 46998 | 45690 | 45290 | 4.28 | 771.8 |
| BBN_4241 | 20830921 | 19075675 | 18316225 | 19407607 | 6.65 | 46340 | 49031 | 46668 | 47346 | 3.10 | 819.8 |
| BBN_11075 | 12451409 | 13597123 | 13782511 | 13277014 | 5.43 | 48344 | 54505 | 54505 | 52451 | 6.78 | 506.3 |
| BBN_16202 | 10874401 | 10231503 | 10801018 | 10635640 | 3.31 | 44106 | 39957 | 41980 | 42015 | 4.94 | 506.3 |
| BBN_19996 | 7803926 | 7647001 | 7442666 | 7631197 | 2.37 | 32334 | 33495 | 32563 | 32797 | 1.88 | 465.4 |
| BBN002.26678 | 7698955 | 7293006 | 6815535 | 7269165 | 6.08 | 37498 | 36973 | 34698 | 36390 | 4.09 | 399.5 |
| BBN002.32884 | 10801018 | 10728130 | 11557696 | 11028948 | 4.17 | 35944 | 33495 | 34454 | 34631 | 3.56 | 636.9 |
| BBN002.32575 | 28636462 | 28443217 | 27871269 | 28316983 | 1.41 | 54122 | 60591 | 57671 | 57461 | 5.64 | 985.6 |

**Abbreviations:** BBN UK: Brain Banks Network. The average copy numbers of mtDNA amplicons (Mt) and nDNA amplicons (N) for each sample were calculated as the mean of three technical replicates. mtDNA-CN was calculated as (Mt/N) × 2. CoV denotes the coefficient of variation, calculated as percentage of (standard deviation/mean) from the three technical replicates per sample; values <20% were considered acceptable.

**Table S3C. Raw data showing mtDNA and nuclear DNA copy numbers obtained by real-time qPCR from human cerebellum**

| **BBN ID** | **Copy number of mtDNA amplicons (hMitoF3-R3)** | | | **Average copy number of mtDNA amplicons**  **(Mt)** | **CoV** | **Copy number of nuclear DNA amplicons (hB2MF1-R1)** | | | **Average copy number of nDNA amplicons**  **(N)** | **CoV** | **MtDNA-CN (copies/cell)** |
| --- | --- | --- | --- | --- | --- | --- | --- | --- | --- | --- | --- |
|  | **Replicate 1** | **Replicate 2** | **Replicate 3** |  |  | **Replicate 1** | **Replicate 2** | **Replicate 3** |  |  |  |
| BBN_22594 | 2741867 | 2575808 | 2666781 | 2661485 | 3.12 | 52522 | 57140 | 54018 | 54560 | 4.32 | 97.6 |
| BBN_23398 | 2091560 | 1937795 | 1710180 | 1913178 | 10.03 | 33983 | 33983 | 32353 | 33439 | 2.81 | 114.4 |
| BBN002.29089 | 1807840 | 1807840 | 1937795 | 1851159 | 4.05 | 28114 | 26392 | 28915 | 27807 | 4.64 | 133.1 |
| BBN002.28414 | 2386443 | 2305033 | 2305033 | 2332169 | 2.02 | 32581 | 29118 | 28312 | 30004 | 7.56 | 155.5 |
| BBN002.28350 | 2838705 | 2666781 | 2799566 | 2768351 | 3.26 | 33745 | 36712 | 40221 | 36893 | 8.79 | 150.1 |
| BBN002.26151 | 2522721 | 2819068 | 2722900 | 2688229 | 5.62 | 38833 | 36455 | 35947 | 37078 | 4.16 | 145.0 |
| BBN002.26311 | 2453636 | 2453636 | 2557989 | 2488420 | 2.42 | 39660 | 39107 | 39107 | 39291 | 0.81 | 126.7 |
| BBN_24666 | 909280 | 1037477 | 902990 | 949915 | 7.99 | 22157 | 18980 | 22470 | 21202 | 9.11 | 89.6 |
| BBN_21801 | 1584453 | 1651840 | 1758332 | 1664875 | 5.27 | 47361 | 44770 | 43223 | 45118 | 4.63 | 73.8 |
| BBN_24370 | 1782915 | 1992356 | 1674934 | 1816735 | 8.88 | 17563 | 17196 | 16372 | 17043 | 3.58 | 213.2 |
| BBN_24243 | 1002085 | 1059309 | 1009065 | 1023486 | 3.05 | 25303 | 22614 | 25126 | 24348 | 6.18 | 84.1 |
| BBN_22991 | 1408083 | 1295541 | 1286578 | 1330067 | 5.09 | 14028 | 13736 | 12985 | 13583 | 3.96 | 195.8 |
| BBN_24297 | 2337313 | 2354176 | 2175184 | 2288891 | 4.32 | 27784 | 29615 | 27784 | 28394 | 3.72 | 161.2 |
| BBN_14408 | 3572063 | 3300474 | 3206919 | 3359819 | 5.65 | 29406 | 33887 | 29826 | 31039 | 7.97 | 216.5 |
| BBN_10993 | 3348270 | 3161141 | 3230056 | 3246489 | 2.92 | 47966 | 46625 | 47627 | 47406 | 1.47 | 137.0 |
| BBN_10214 | 1540411 | 1740652 | 1691311 | 1657458 | 6.29 | 19628 | 20337 | 18415 | 19460 | 4.99 | 170.3 |
| BBN_10209 | 1830486 | 1753210 | 1765859 | 1783185 | 2.32 | 22942 | 24454 | 23436 | 23611 | 3.27 | 151.0 |
| BBN002.36235 | 2113527 | 2083357 | 2009802 | 2068895 | 2.58 | 23270 | 20627 | 23270 | 22389 | 6.82 | 184.8 |
| BBN002.29056 | 2801275 | 3037422 | 3442128 | 3093608 | 10.48 | 36693 | 36950 | 39346 | 37663 | 3.89 | 164.3 |
| BBN002.28762 | 761676 | 750550 | 819831 | 777352 | 4.79 | 42192 | 38801 | 41607 | 40866 | 4.44 | 38.0 |
| BBN002.29910 | 3391846 | 3317798 | 3293477 | 3334374 | 1.54 | 45879 | 44615 | 42192 | 44229 | 4.24 | 150.8 |
| BBN002.31486 | 1882800 | 1939036 | 1953355 | 1925064 | 1.94 | 31032 | 35434 | 33509 | 33325 | 6.62 | 115.5 |
| BBN002.32545 | 2400228 | 2680300 | 2564549 | 2548359 | 5.52 | 37997 | 35188 | 33981 | 35722 | 5.77 | 142.7 |
| BBN002.32818 | 4355918 | 4137252 | 4586142 | 4359771 | 5.15 | 44615 | 40179 | 38531 | 41109 | 7.65 | 212.1 |
| BBN002.35184 | 1444674 | 1362093 | 1322590 | 1376452 | 4.53 | 30816 | 29968 | 30602 | 30462 | 1.45 | 90.4 |
| BBN002.35898 | 1014824 | 978169 | 1029868 | 1007620 | 2.64 | 39900 | 42487 | 44305 | 42231 | 5.24 | 47.7 |
| BBN002.36128 | 1488492 | 1408083 | 1498861 | 1465145 | 3.39 | 40858 | 46372 | 51896 | 46375 | 11.90 | 63.2 |
| BBN_23396 | 2760967 | 2780200 | 2741867 | 2761011 | 0.69 | 47361 | 45404 | 45725 | 46163 | 2.27 | 119.6 |
| BBN_16191 | 1051981 | 860160 | 1051981 | 988041 | 11.21 | 24062 | 21086 | 23712 | 22953 | 7.09 | 86.1 |
| BBN_10192 | 1350640 | 1379063 | 1242689 | 1324130 | 5.43 | 31797 | 30652 | 31565 | 31338 | 1.93 | 84.5 |
| BBN_9985 | 470213 | 473488 | 454172 | 465958 | 2.22 | 17045 | 16431 | 16797 | 16758 | 1.84 | 55.6 |
| BBN_4570 | 2722900 | 2419806 | 2648333 | 2597013 | 6.08 | 41105 | 34979 | 38762 | 38282 | 8.07 | 135.7 |
| BBN_16694 | 4463786 | 3921986 | 4463786 | 4283186 | 7.30 | 27588 | 27982 | 27007 | 27525 | 1.78 | 311.2 |
| BBN002.26310 | 1924956 | 2038907 | 1778599 | 1914154 | 6.82 | 31567 | 28583 | 28992 | 29714 | 5.44 | 128.8 |
| BBN002.30139 | 3893893 | 4154155 | 3950283 | 3999443 | 3.42 | 44683 | 45321 | 42218 | 44074 | 3.72 | 181.5 |
| BBN002.32840 | 1155457 | 1518422 | 1402974 | 1358951 | 13.65 | 10819 | 10442 | 11450 | 10904 | 4.67 | 249.3 |
| BBN002.33675 | 2511525 | 2584793 | 2622225 | 2572848 | 2.19 | 28992 | 28583 | 29406 | 28994 | 1.42 | 177.5 |
| BBN002.35541 | 5154005 | 5080433 | 5007911 | 5080783 | 1.44 | 26816 | 29406 | 24804 | 27009 | 8.54 | 376.2 |
| BBN002.36344 | 1509301 | 1457814 | 1457814 | 1474977 | 2.02 | 16841 | 19385 | 21092 | 19106 | 11.20 | 154.4 |
| BBN_9958 | 4811085 | 4912327 | 4646962 | 4790125 | 2.80 | 76410 | 70225 | 68759 | 71798 | 5.66 | 133.4 |
| BBN_4194 | 4101127 | 3933821 | 3852745 | 3962564 | 3.20 | 53377 | 48371 | 51896 | 51215 | 5.02 | 154.7 |
| BBN_16192 | 3644619 | 3670007 | 3594368 | 3636331 | 1.06 | 27722 | 26392 | 27917 | 27343 | 3.04 | 266.0 |
| BBN002.26316 | 5265444 | 5156924 | 5050640 | 5157669 | 2.08 | 50813 | 54516 | 53754 | 53027 | 3.69 | 194.5 |
| BBN002.32584 | 1200296 | 1225555 | 1167426 | 1197759 | 2.43 | 20944 | 22629 | 20944 | 21506 | 4.52 | 111.4 |
| BBN002.32590 | 1488492 | 1398342 | 1562607 | 1483147 | 5.55 | 25503 | 25865 | 24622 | 25330 | 2.52 | 117.1 |
| BBN_16307 | 2062722 | 2077091 | 1884728 | 2008180 | 5.34 | 39446 | 35496 | 37287 | 37409 | 5.29 | 107.4 |
| BBN002.35480 | 5604901 | 5966243 | 5643945 | 5738363 | 3.46 | 35496 | 35247 | 37550 | 36098 | 3.50 | 317.9 |
| BBN_16429 | 4044582 | 5121250 | 4878345 | 4681392 | 12.06 | 21542 | 20944 | 19659 | 20715 | 4.64 | 452.0 |
| BBN002.37301 | 1167426 | 1295541 | 1191993 | 1218320 | 5.58 | 8402 | 7456 | 8641 | 8166 | 7.67 | 298.4 |
| BBN_23793 | 3307084 | 3284207 | 3063958 | 3218416 | 4.17 | 33967 | 35236 | 41105 | 36769 | 10.36 | 175.1 |
| BBN002.28760 | 2048453 | 2150450 | 1937795 | 2045566 | 5.20 | 31565 | 29766 | 25330 | 28887 | 11.11 | 141.6 |
| BBN002.33690 | 1782915 | 1795334 | 1884728 | 1820992 | 3.05 | 29985 | 29985 | 27864 | 29278 | 4.18 | 124.4 |
| BBN002.30168 | 5413699 | 5643945 | 4679332 | 5245659 | 9.60 | 45217 | 41407 | 42640 | 43088 | 4.51 | 243.5 |
| BBN_9851 | 1143366 | 884379 | 954555 | 994100 | 13.47 | 14294 | 14612 | 12994 | 13966 | 6.14 | 142.4 |
| BBN002.30072 | 3284207 | 3799625 | 3933821 | 3672551 | 9.34 | 48659 | 46906 | 46906 | 47491 | 2.13 | 154.7 |
| BBN002.36940 | 2353539 | 1951293 | 2273252 | 2192695 | 9.71 | 29766 | 29118 | 31565 | 30150 | 4.20 | 145.5 |
| BBN_4195 | 1858742 | 1663347 | 1551798 | 1691296 | 9.19 | 28276 | 26861 | 23538 | 26225 | 9.27 | 129.0 |
| BBN_9884 | 6350880 | 6575182 | 7047831 | 6657964 | 5.34 | 57671 | 56467 | 55678 | 56605 | 1.77 | 235.2 |
| BBN_9927 | 4158462 | 4158462 | 4519704 | 4278876 | 4.87 | 11682 | 11682 | 13073 | 12146 | 6.61 | 704.6 |
| BBN_4241 | 1183747 | 1051981 | 1104361 | 1113363 | 5.96 | 29356 | 27945 | 26231 | 27844 | 5.62 | 80.0 |
| BBN_11075 | 3238926 | 3307084 | 3799625 | 3448545 | 8.87 | 33085 | 30407 | 31055 | 31516 | 4.43 | 218.8 |
| BBN_16202 | 5413699 | 5527623 | 4711928 | 5217750 | 8.47 | 32394 | 30621 | 33790 | 32268 | 4.92 | 323.4 |
| BBN_19996 | 4044582 | 4072757 | 4044582 | 4053974 | 0.40 | 43835 | 44144 | 47696 | 45225 | 4.74 | 179.3 |
| BBN002.26678 | 1734089 | 1734089 | 1833115 | 1767098 | 3.24 | 40287 | 38082 | 39446 | 39272 | 2.83 | 90.0 |
| BBN002.32884 | 6807405 | 7146362 | 7296748 | 7083505 | 3.54 | 50813 | 44456 | 51533 | 48934 | 7.96 | 289.5 |
| BBN002.32575 | 2741867 | 2666781 | 2470727 | 2626459 | 5.33 | 38622 | 34755 | 32853 | 35410 | 8.30 | 148.3 |

**Abbreviations:** BBN UK: Brain Banks Network. The average copy numbers of mtDNA amplicons (Mt) and nDNA amplicons (N) for each sample were calculated as the mean of three technical replicates. mtDNA-CN was calculated as (Mt/N) × 2. CoV denotes the coefficient of variation, calculated as percentage of (standard deviation/mean) from the three technical replicates per sample; values <20% were considered acceptable.

**Table S4. Sex distribution and sex-stratified mtDNA-CN analyses**

**Part A. Sex distribution across study groups**

| **Group** | **Male** | **Female** | **Total** | **% Female** |
| --- | --- | --- | --- | --- |
| CC | 10 | 10 | 20 | 50.0 |
| CC+D | 8 | 10 | 18 | 55.6 |
| AD | 8 | 12 | 20 | 60.0 |
| AD+D | 10 | 9 | 19 | 47.4 |
| Total | 36 | 41 | 77 | 53.2 |

Sex distribution did not differ significantly across study groups (χ² = 0.75, df = 3, P = 0.861).

**Part B. Association between sex and mtDNA-CN across brain regions and study groups**

| **Brain region** | **Group** | **MtDNA-CN in males**  **Mean ± SD (n)** | **MtDNA-CN in females**  **Mean ± SD (n)** | **P-value** | **Interpretation** |
| --- | --- | --- | --- | --- | --- |
| Hippocampus | Overall | 396.4 ± 206.2  (36) | 441.8 ± 190.1  (41) | 0.1486 | Not significant |
|  | CC | 300.7 ± 82.9  (10) | 485.3 ± 187.9  (10) | 0.0312* | Nominal; not significant after correction |
|  | CC+D | 560.1 ± 309.4  (8) | 507.2 ± 211.8  (10) | 0.8968 | Not significant |
|  | AD | 296.0 ± 108.8  (8) | 335.5 ± 183.9  (12) | 0.6784 | Not significant |
|  | AD+D | 441.4 ± 172.2  (10) | 462.7 ± 137.0  (9) | 0.7133 | Not significant |

| Amygdala | Overall | 479.0 ± 242.8  (36) | 524.5 ± 228.7  (41) | 0.2724 | Not significant |
| --- | --- | --- | --- | --- | --- |
|  | CC | 529.0 ± 310.6  (10) | 508.5 ± 228.0  (10) | 0.9698 | Not significant |
|  | CC+D | 596.5 ± 320.1  (8) | 600.7 ± 285.8  (10) | 0.7618 | Not significant |
|  | AD | 318.0 ± 93.2  (8) | 389.4 ± 116.4  (12) | 0.1569 | Not significant |
|  | AD+D | 464.0 ± 88.9  (10) | 642.1 ± 206.4  (9) | 0.079 | Not significant |
| Cerebellum | Overall | 165.5 ± 78.8  (31) | 153.8 ± 65.1  (33) | 0.643 | Not significant |
|  | CC | 146.6 ± 39.2  (10) | 141.8 ± 48.7  (8) | 0.9654 | Not significant |
|  | CC+D | 245.4 ± 95.3  (6) | 180.7 ± 74.6  (10) | 0.1806 | Not significant |
|  | AD | 101.6 ± 60.7  (6) | 112.1 ± 44.4  (8) | 0.662 | Not significant |
|  | AD+D | 175.9 ± 72.0  (9) | 176.8 ± 69.5  (7) | 0.6806 | Not significant |

**Abbreviations:** AD: Alzheimer's disease; AD+D: Alzheimer's disease with diabetes; CC: Cognitive controls; CC+D: Cognitive controls with diabetes.

Data are shown as mean ± SD. P-values compare mtDNA-CN between males and females using Mann–Whitney U tests. Correction for multiple testing was applied across the three brain regions, with corrected significance defined as P < 0.0167.

*Nominally significant (P = 0.031) but does not survive the regional multiple-comparison threshold (P < 0.0167).

**Table S5. Principal component analysis of mtDNA-CN across three brain regions**

| **(A) Variance explained and component loadings for the overall PCA** | | | | | | |
| --- | --- | --- | --- | --- | --- | --- |
| **Component** | **Variance**  **explained (%)** | **Loading: Hippocampus** | | **Loading: Amygdala** | **Loading: Cerebellum** |  |
| PC1 | 60.7 | 0.644 | | 0.531 | 0.551 |  |
| PC2 | 25.3 | -0.031 | | 0.738 | -0.674 |  |
| PC3 | 13.9 | 0.765 | | -0.417 | -0.491 |  |
| **(B) PC Scores by disease group (Mean ± SD)** | | | | | | |
| **Group** | **N** | **PC1** | | **PC2** | **PC3** |  |
| Entire dataset | 64 | −0.00 ± 1.36 | | −0.00 ± 0.88 | +0.00 ± 0.65 |  |
| CC | 18 | −0.30 ± 0.93 | | +0.20 ± 1.04 | −0.18 ± 0.52 |  |
| CC+D | 16 | +1.02 ± 1.66 | | −0.17 ± 0.97 | +0.08 ± 0.85 |  |
| AD | 14 | −1.22 ± 0.57 | | −0.01 ± 0.58 | +0.13 ± 0.60 |  |
| AD+D | 16 | +0.39 ± 1.00 | | −0.04 ± 0.84 | +0.01 ± 0.61 |  |
| Kruskal-Wallis test | | H = 26.13, p < 0.0001 | H = 0.41, p = 0.939 | | H = 2.20, p = 0.532 |  |

| **(C) PC1 loadings by disease group** | | | | | |
| --- | --- | --- | --- | --- | --- |
| **Group** | **N** | **PC1 variance explained (%)** | **Loading: Hippocampus** | **Loading: Amygdala** | **Loading: Cerebellum** |
| CC | 18 | 47.9 | 0.630 | 0.742 | -0.230 |
| CC+D | 16 | 61.5 | 0.619 | 0.532 | 0.578 |
| AD | 14 | 47.0 | 0.700 | 0.569 | -0.432 |
| AD+D | 16 | 49.3 | 0.690 | 0.463 | 0.556 |

**Abbreviations:** AD: Alzheimer's disease; AD+D: Alzheimer's disease with diabetes; CC: Cognitive controls; CC+D: Cognitive controls with diabetes. **(A)** Variance explained and component loadings for PC1, PC2, and PC3 from PCA performed on standardised (z-scored) mtDNA-CN values across hippocampus, amygdala, and cerebellum (n = 64). PC1 captures the shared co-variation across all three regions, PC2 captures a contrast between amygdala and cerebellum, and PC3 captures a contrast between hippocampus and the other two regions. **(B)** Mean ± SD of PC1, PC2, and PC3 scores for the overall dataset and each disease group. Group differences were assessed using the Kruskal–Wallis H test. Only PC1 differed significantly between groups (H = 26.13, p < 0.0001), with CC+D showing the highest scores (+1.02 ± 1.66) and AD the lowest (−1.22 ± 0.57). (C) PC1 loadings from PCA performed separately within each disease group using standardised (z-scored) mtDNA-CN values across hippocampus, amygdala, and cerebellum (n = 64). In CC+D and AD+D, hippocampus, amygdala, and cerebellum all loaded positively on PC1. In CC and AD, hippocampus and amygdala loaded positively whereas cerebellum loaded in the opposite direction, suggesting that cerebellum was more distinct in these groups.

**Table S6.** **Intraclass correlation coefficient (ICC) and Kendall's coefficient of concordance (W) for mtDNA-CN in three brain regions.**

| **(A) Intraclass Correlation Coefficient (ICC 3,1)** | | | | | | | | | |
| --- | --- | --- | --- | --- | --- | --- | --- | --- | --- |
| **Group** | **N** | | **ICC**  **(raw data)** | | **P-value** | | **ICC**  **(z-scored data)** | | **p-value** |
| Overall | 64 | | 0.331 | | < 0.001 | | 0.406 | | < 0.001 |
| CC | 18 | | 0.139 | | 0.16 | | 0.102 | | 0.227 |
| CC+D | 16 | | 0.331 | | 0.017 | | 0.423 | | 0.003 |
| AD | 14 | | 0.092 | | 0.273 | | 0.022 | | 0.425 |
| AD+D | 16 | | 0.2 | | 0.094 | | 0.229 | | 0.067 |
| **(B) Kendall's Coefficient of Concordance (W)** | | | | | | | | | |
| **Group** | | **n** | | **Kendall's W** | | **χ²** | | **p-value** | |
| Overall | | 64 | | 0.708 | | 90.66 | | < 0.001 | |
| CC | | 18 | | 0.799 | | 28.78 | | < 0.001 | |
| CC+D | | 16 | | 0.660 | | 21.12 | | < 0.001 | |
| AD | | 14 | | 0.597 | | 16.71 | | < 0.001 | |
| AD+D | | 16 | | 0.785 | | 25.12 | | < 0.001 | |

**Abbreviations:** AD: Alzheimer's disease; AD+D: Alzheimer's disease with diabetes; CC: Cognitive controls; CC+D: Cognitive controls with diabetes. **(A)** Intraclass correlation coefficient (ICC 3,1) values showing the degree of absolute agreement in mtDNA‑CN across hippocampus, amygdala, and cerebellum within individuals. ICCs were calculated using both raw mtDNA‑CN values and z‑scored data. Corresponding p-values indicate statistical significance. **(B)** Kendall’s coefficient of concordance (W) assessing rank‑order agreement of mtDNA‑CN across the three brain regions within each group. Associated chi‑square (χ²) statistics and p‑values are shown.

| **Table S7. Summary of published studies evaluating mitochondrial DNA copy number in Alzheimer's disease using human post-mortem brain tissue** | | | | |
| --- | --- | --- | --- | --- |
| **Brain region** | **Technique** | **MtDNA-CN calculations** | **Results** | **Reference** |
| Dorsolateral prefrontal cortex (DLPFC, n=367) from ROSMAP cohort | Estimated mtDNA-CN (Whole genome sequencing) | (Average mitochondrial coverage ÷ average autosomal coverage) × 2 | Lower mtDNA-CN in DLPFC linked to higher odds of dementia (OR=0.61, p=0.0008), worse cognition (β=0.32, p=1.7×10⁻⁵), faster decline, more tau tangles (p=0.0007). No association with amyloid. | Lynch MT, Taub MA, Farfel JM, et al. Evaluating genomic signatures of aging in brain tissue as it relates to Alzheimer's disease. *Sci Rep*. 2023;13(1):14747. DOI: [10.1038/s41598-023-41400-1](https://doi.org/10.1038/s41598-023-41400-1) |
| DLPFC (n=461), PCC (n=68), TCX (n=168), FP (n=288), CBE (n=265); cohorts: ROSMAP, MSBB, Mayo, Banner | Estimated mtDNA-CN (Whole genome sequencing) | (Average mitochondrial coverage ÷ average autosomal coverage) × 2 | Higher mtDNA-CN associated with reduced odds of neuropathological AD (OR=0.64, p=0.002) and lower tau tangle burden (OR=0.61, p=7.8×10⁻⁵). No association with amyloid plaques. Strongest associations in DLPFC and PCC. No associations in cerebellum. | Harerimana NV, Paliwali D, Romero-Molina C, et al. The role of mitochondrial genome abundance in Alzheimer's disease. *Alzheimers Dement*. 2023;19(5):2069-2083. DOI: [10.1002/alz.12812](https://doi.org/10.1002/alz.12812) |
| DLPFC (n=454), PCC (n=66), TCX (n=262), FP (n=337), CBE (n=242); cohorts: ROSMAP, MSBB, Mayo | Estimated mtDNA-CN (Whole genome sequencing) | Median sequence coverages of the autosomal chromosomes covnuc and of the mitochondrial genome covmt  (covmt/covnuc) X 2 | 7–14% decrease in mtDNA-CN in AD cases compared to controls in cortical regions (DLPFC, PCC, TCX, FP). No significant changes in cerebellum. | Klein HU, Trumpff C, Yang HS, et al. Characterization of mitochondrial DNA quantity and quality in the human aged and Alzheimer's disease brain. *Mol Neurodegener*. 2021;16(1):75.  DOI: [10.1186/s13024-021-00495-8](https://doi.org/10.1186/s13024-021-00495-8) |
| 282 samples from cerebellum (87.3%), cerebral cortex (6.5%), other (6.2%) from UK MRC Brain Tissue Resource | Estimated mtDNA-CN (Exome Sequencing) | Mean mtDNA read depth ÷ mean exome read depth | mtDNA-CN decreased in AD brains compared to controls. Regional variation not reported. | Wei W, Keogh MJ, Wilson I, et al. Mitochondrial DNA point mutations and relative copy number in 1363 disease and control human brains. *Acta Neuropathol Commun*. 2017;5(1):13. DOI: [10.1186/s40478-016-0404-6](https://doi.org/10.1186/s40478-016-0404-6) |
| Frontal cortex (n=74), parietal cortex (n=72), cerebellum (n=54) | Absolute quantification (qPCR) | (mtDNA copies ÷ nuclear hB2M copies) × 2 | mtDNA-CN increased in diabetes across all brain regions, irrespective of cognitive status. In parietal cortex, non-diabetic AD had decreased mtDNA-CN, reduced MAP2 (neurons), and increased GFAP (astrocytes); changes absent in diabetic AD. | Thubron EB, Rosa HS, Hodges A, et al. Regional mitochondrial DNA and cell-type changes in post-mortem brains of non-diabetic Alzheimer's disease are not present in diabetic Alzheimer's disease. *Sci Rep*. 2019;9(1):11386. DOI: [10.1038/s41598-019-47783-4](https://doi.org/10.1038/s41598-019-47783-4) |
| Temporal cortex (n=10), cerebellum (n=10) | ddPCR | Relative amplification between mitochondrial (Walker) and nuclear (RRP30) loci | mtDNA-CN decreased in temporal cortex, unchanged in cerebellum. | Soltys DT, Pereira CPM, Rowies FT, et al. Lower mitochondrial DNA content but not increased mutagenesis associates with decreased base excision repair activity in brains of AD subjects. *Neurobiol Aging*. 2019; 73:161-170. DOI: [10.1016/j.neurobiolaging.2018.09.015](https://doi.org/10.1016/j.neurobiolaging.2018.09.015) |
| Hippocampus (10 AD, 9 controls) | Absolute quantification (qPCR) | (mtDNA copies ÷nuclear B2M copies) × 2, averaged across 4 mtDNA loci (12S rRNA, ND2, COX III, ND4) | Decreased mtDNA-CN in post-mortem AD hippocampal pyramidal neurons | Rice AC, Keeney PM, Algarzae NK, Ladd AC, Thomas RR, Bennett JP Jr. Mitochondrial DNA copy numbers in pyramidal neurons are decreased and mitochondrial biogenesis transcriptome signaling is disrupted in Alzheimer's disease hippocampi. *J Alzheimers Dis*. 2014;40(2):319-330.  DOI: [10.3233/JAD-131715](https://doi.org/10.3233/jad-131715) |
| Frontal cortex, hippocampus and cerebellum (AD=12, controls=7) | qPCR | Relative mtND2 ÷ r18S | No significant differences in mtDNA-CN between AD and controls in frontal cortex, cerebellum and hippocampus. | Rodríguez-Santiago B, Casademont J, Nunes V. Is mitochondrial DNA depletion involved in Alzheimer's disease?. *Eur J Hum Genet*. 2001;9(4):279-285. DOI: [10.1038/sj.ejhg.5200629](https://doi.org/10.1038/sj.ejhg.5200629) |

**Abbreviations:** AD: Alzheimer’s disease; CBE: cerebellum; covmt: mitochondrial genome coverage; covnuc : autosomal genome coverage; DLPFC: dorsolateral prefrontal cortex; FP: frontal pole; GFAP: glial fibrillary acidic protein; MAP2: microtubule-associated protein 2; MSBB: Mount Sinai Brain Bank; mtDNA-CN: mitochondrial DNA copy number; ND: NADH dehydrogenase subunit; PCC: posterior cingulate cortex; qPCR: quantitative polymerase chain reaction; RRP30: ribonuclease P protein subunit 30; ROSMAP: Religious Orders Study and Memory and Aging Project; TCX: temporal cortex; UK MRC: UK Medical Research Council.

**Table S8A. Medication history for diabetic subjects^#^**

| **BBN ID** | **Group** | **Sex** | **Age at death (years)** | **Year of death** | **Diabetes diagnosis year** | **Duration of diabetes (years)** | **Anti-diabetic treatment** |  |
| --- | --- | --- | --- | --- | --- | --- | --- | --- |
| BBN002.26310 | CC+D | Female | 93 | – | – | – | None documented |  |
| BBN002.26316 | CC+D | Female | 95 | 2015 | – | – | Insulin glargine (Lantus) |  |
| BBN002.30139 | CC+D | Female | 47 | 2017 | – | – | Insulin aspart (NovoRapid); Insulin detemir (Levemir) |  |
| BBN002.32584 | CC+D | Female | 98 | 2018 | – | – | None documented |  |
| BBN002.32590 | CC+D | Female | 81 | 2018 | – | – | None documented |  |
| BBN002.32840 | CC+D | Female | 88 | 2018 | 2010 | 8 | Linagliptin; Metformin |  |
| BBN002.33675 | CC+D | Male | 90 | 2018 | 2012 | 6 | None documented |  |
| BBN002.33764 | CC+D | Male | 73 | 2018 | – | – | Insulin detemir (Levemir) |  |
| BBN002.35480 | CC+D | Female | 79 | 2019 | – | – | None documented |  |
| BBN002.35541 | CC+D | Male | 75 | 2019 | – | – | Insulin aspart (NovoRapid); Insulin glargine (Lantus); Metformin |  |
| BBN002.36344 | CC+D | Male | 79 | 2020 | – | – | Metformin; Pioglitazone; Sitagliptin |  |
| BBN002.37301 | CC+D | Male | 62 | 2022 | – | – | Medication history not available |  |
| BBN_16192 | CC+D | Female | 81 | 2013 | – | – | Metformin |  |
| BBN_16307 | CC+D | Female | 55 | 2008 | 2003 | 5 | Gliclazide; Metformin |  |
| BBN_16429 | CC+D | Male | 68 | 2000 | – | – | Medication history not available |  |
| BBN_16694 | CC+D | Male | 75 | 1994 | – | – | Medication history not available |  |
| BBN_4194 | CC+D | Male | 97 | 2011 | 1993 | 18 | Tolbutamide |  |
| BBN_9958 | CC+D | Female | 84 | 2011 | – | – | Diet-controlled; later oral diabetes medication mentioned, drug not specified |  |
| BBN002.26678 | AD+D | Male | 86 | 2015 | 2002 | 13 | None documented |  |
| BBN002.28760 | AD+D | Male | 76 | 2014 | – | – | Insulin glargine (Lantus) |  |
| BBN002.30072 | AD+D | Female | 82 | 2017 | – | – | Metformin |  |
| BBN002.30168 | AD+D | Female | 68 | 2017 | – | – | Medication history not available |  |
| BBN002.32575 | AD+D | Female | 89 | 2018 | – | – | Gliclazide; Metformin; Saxagliptin |  |
| BBN002.32884 | AD+D | Female | 82 | 2018 | – | – | Insulin degludec (Tresiba) |  |
| BBN002.33690 | AD+D | Male | 84 | 2018 | – | – | Metformin |  |
| BBN002.33768 | AD+D | Female | 92 | 2018 | – | – | None documented |  |
| BBN002.36940 | AD+D | Male | 81 | 2021 | – | – | Alogliptin; Gliclazide; Metformin; Saxagliptin |  |
| BBN_11075 | AD+D | Male | 78 | 2012 | – | – | Medication history not available |  |
| BBN_16202 | AD+D | Male | 67 | 2013 | 2004 | 9 | None documented |  |
| BBN_19996 | AD+D | Female | 75 | 2013 | – | – | Gliclazide; Metformin |  |
| BBN_23793 | AD+D | Male | 84 | 2014 | – | – | None documented |  |
| BBN_4195 | AD+D | Male | 83 | 2011 | 2001 | 10 | Diet-controlled |  |
| BBN_4241 | AD+D | Female | 94 | 2012 | – | – | Medication history not available |  |
| BBN_9845 | AD+D | Male | 81 | 2007 | 2004 | 3 | Insulin glargine (Lantus); Metformin |  |
| BBN_9851 | AD+D | Female | 82 | 2007 | 1990 | 17 | Diet-controlled |  |
| BBN_9884 | AD+D | Male | 74 | 2008 | 1999 | 9 | Gliclazide |  |
| BBN_9927 | AD+D | Female | 90 | 2009 | – | – | None documented |  |

**Abbreviations:** AD+D: Alzheimer's disease with diabetes; CC+D: Cognitive controls with diabetes.

^#^Medication history was available for 31 of 37 diabetic subjects. Of these, antidiabetic therapy was documented or mentioned in 19 cases, including one subject for whom oral diabetes medication was mentioned but the drug was not specified., 2 were diet-controlled, 10 had medication history available but no anti-diabetic drugs were documented. For the remaining 6 diabetic subjects, medication history was not available. One diet-controlled subject had later oral diabetes medication mentioned, but the drug was not specified. Anti-diabetic treatments were defined as documented insulin preparations or oral glucose-lowering medications, including metformin, gliclazide, tolbutamide, pioglitazone, sitagliptin, saxagliptin, alogliptin, linagliptin, and insulin preparations including aspart, detemir, glargine and degludec.

**Table S8B. Exploratory analysis of mtDNA-CN according to antidiabetic medication history in diabetic subjects.**

| 1. **Comparison of mtDNA-CN in diabetic subjects with antidiabetic therapy versus no recorded antidiabetic therapy/diet control** | | | | |
| --- | --- | --- | --- | --- |
| **Brain region** | **mtDNA-CN in cases with anti-diabetic therapy**  **mean ± SD (n)** | **mtDNA-CN in cases with no recorded anti-diabetic therapy/diet-controlled**  **mean ± SD (n)** | **P value** | **Interpretation** |
| **Hippocampus** | 454.0 ± 195.1 (n = 19) | 470.2 ± 172.9 (n = 12) | 0.584 | Not significant |
| **Amygdala** | 619.3 ± 240.9 (n = 19) | 539.4 ± 230.4 (n = 12) | 0.301 | Not significant |
| **Cerebellum** | 190.3 ± 70.8 (n = 17) | 171.3 ± 83.2 (n = 10) | 0.219 | Not significant |

| 2. **Comparison of mtDNA-CN in metformin users versus non-metformin diabetic subjects** | | | | |
| --- | --- | --- | --- | --- |
| **Brain region** | **mtDNA-CN in metformin users, mean ± SD (n)** | **mtDNA-CN in non-metformin diabetic subjects, mean ± SD (n)** | **P value** | **Interpretation** |
| **Hippocampus** | 459.7 ± 188.9 (n = 11) | 502.9 ± 218.4 (n = 26) | 0.7272 | Not significant |
| **Amygdala** | 572.1 ± 249.5 (n = 11) | 573.3 ± 237.9 (n = 26) | 0.855 | Not significant |
| **Cerebellum** | 190.6 ± 82.6 (n = 10) | 190.7 ± 77.7 (n = 22) | 1.000 | Not significant |
| **3. Exploratory comparison across medication categories** | | | | |
| **Hippocampus** | Antidiabetic therapy (n=19); no antidiabetic therapy (n=10); diet-controlled (n=2); no medication data (n=6) | Kruskal-Wallis | 0.184 | Not significant |
| **Amygdala** | Antidiabetic therapy (n=19); no antidiabetic therapy (n=10); diet-controlled (n=2); no medication data (n=6) | Kruskal-Wallis | 0.446 | Not significant |
| **Cerebellum** | Antidiabetic therapy (n=17); no antidiabetic therapy (n=8); diet-controlled (n=2); no medication data (n=5) | Kruskal-Wallis | 0.441 | Not significant |

Data are shown as mean ± SD. Mann-Whitney U tests compared mtDNA-CN between diabetic subjects with recorded antidiabetic therapy and those with no recorded antidiabetic therapy/diet control, and between metformin users and non-metformin diabetic subjects. Kruskal-Wallis tests were used for exploratory comparisons across medication categories. Two cerebellum outliers were excluded from cerebellum analyses, resulting in lower n values for cerebellum comparisons. Analyses were exploratory due to small subgroup sizes and incomplete retrospective medication histories. One subject classified in the antidiabetic therapy group had oral diabetes medication mentioned, but the drug was not specified.
